# Comprehensive full genome analysis of norovirus strains from eastern India, 2017–2021

**DOI:** 10.1186/s13099-023-00594-5

**Published:** 2024-01-18

**Authors:** Mahadeb Lo, Yen Hai Doan, Suvrotoa Mitra, Ritubrita Saha, Shin-ichi Miyoshi, Kei Kitahara, Shanta Dutta, Tomoichiro Oka, Mamta Chawla-Sarkar

**Affiliations:** 1 Division of Virology, ICMR-National Institute of Cholera and Enteric Diseases, P-33, C.I.T. Rd, Scheme‐XM, Beliaghata, Kolkata, 700010 West Bengal India; 2 Center for Emergency Preparedness and Response, National Institute of Infectious Diseases, Gakuen 4-7-1, Musashi-Murayama, Tokyo Japan; 3 Collaborative Research Center of Okayama University for Infectious Diseases in India, ICMR-National Institute of Cholera and Enteric Diseases, Kolkata, India; 4 Graduate School of Medicine, Dentistry and Pharmaceutical Sciences, Okayama University, Okayama, Japan; 5 Regional Virus Research and Diagnostic Laboratory, ICMR-National Institute of Cholera and Enteric Diseases, Beliaghata, Kolkata, West Bengal India; 6 Department of Virology II, National Institute of Infectious Diseases, Gakuen 4-7-1, Musashi-Murayama, Tokyo Japan

## Abstract

**Background:**

Worldwide, noroviruses are the leading cause of acute gastroenteritis (AGE) in people of all age groups. In India, norovirus rates between 1.4 to 44.4% have been reported. Only a very few complete norovirus genome sequences from India have been reported.

**Objective:**

To perform full genome sequencing of noroviruses circulating in India during 2017–2021, identify circulating genotypes, assess evolution including detection of recombination events.

**Methodology:**

Forty-five archived norovirus-positive samples collected between October 2017 to July 2021 from patients with AGE from two hospitals in Kolkata, India were processed for full genome sequencing. Phylogenetic analysis, recombination breakpoint analysis and comprehensive mutation analysis were also performed.

**Results:**

Full genome analysis of norovirus sequences revealed that strains belonging to genogroup (G)I were genotyped as GI.3[P13]. Among the different norovirus capsid-polymerase combinations, GII.3[P16], GII.4 Sydney[P16], GII.4 Sydney[P31], GII.13[P16], GII.16[P16] and GII.17 were identified. Phylogenetic analysis confirmed phylogenetic relatedness with previously reported norovirus strains and all viruses were analyzed by Simplot. GII[P16] viruses with multiple residue mutations within the non-structural region were detected among circulating GII.4 and GII.3 strains. Comprehensive mutation analysis and selection pressure analysis of GII[P16] viruses showed positive as well as negative selection sites. A GII.17 strain (NICED-BCH-11889) had an untypeable polymerase type, closely related to GII[P38].

**Conclusion:**

This study highlights the circulation of diverse norovirus strains in eastern India. These findings are important for understanding norovirus epidemiology in India and may have implications for future vaccine development.

**Supplementary Information:**

The online version contains supplementary material available at 10.1186/s13099-023-00594-5.

## Introduction

Noroviruses are a major cause of acute gastroenteritis (AGE) in people across all age groups. Worldwide, about 18% of AGE cases are associated with norovirus with an estimated 200,000 deaths annually [1, 2]. Norovirus outbreaks have a significant financial impact estimated to be more than $64 billion yearly [3, 4]. In spite of huge morbidity and mortality across the world, no antivirals or vaccines against norovirus have been licensed yet.

Noroviruses are genetically diverse group of viruses belonging to the family *Caliciviridae.* They contain a single stranded positive sense RNA genome of ∼ 7.6 kb long which is comprised of three open reading frames (ORFs). ORF1 encodes non-structural polyprotein which is proteolytically cleaved to produce six non-structural proteins including viral RNA dependent RNA polymerase (NS7). ORF2 and ORF3 encode major capsid protein (Viral Protein 1; VP1) and minor capsid protein (Viral protein 2; VP2) respectively [5]. Based on amino acid diversity of VP1, ten norovirus genogroups (GI-GX) have been identified. Each genogroup comprises multiple genotypes. Humans can be infected by more than 36 different genotypes from 5 different genogroups (GI, GII, GIV, GVIII and GIX) [6]. Of these, GII viruses are most frequently detected GII.4 viruses are the predominant genotype associated with 50–70% of all norovirus outbreaks [7]. In addition to VP1 diversity, point mutations and recombination further contribute to the greater genetic diversity of noroviruses. One of the major recombination hotspots is located at the junction of ORF1 and ORF2, thus dual typing has become the standard method to type noroviruses [6].

Reported rates of norovirus infections in different parts of India vary widely. In northern and southern India, rates ranged between 1.4 and 44.4% [8, 9], while in western India, a rate of 10.7% was reported [10, 11]. Previous studies from eastern India reported a prevalence of 3.1–6.0% among children with AGE [12–16]. Most genotype reports are based on a frequently used small region of the genome and full genome norovirus sequences from India are lacking. In the present study, we performed full genome sequencing of noroviruses circulating during 2017–2021 to comprehensively characterize in eastern India.

## Materials and methods

### Study site and ethical clearance

Kolkata is among the 30 megacities in the world and 3rd largest populous metropolitan in Eastern part of India. During this study, fecal specimens from people of all ages were collected from two tertiary care referral hospitals (Infectious Disease and Beliaghata General Hospital (IDH) and Dr. B.C. Roy Post-Graduate Institute of Pediatric Sciences (BCPGIPS)) (Additional file 6: Table S1). The study was approved by institutional ethics committees of participating institutes.

### Sample curation and processing

A total of 45 archived norovirus-positive specimens collected between October 2017 to July 2021 were selected for whole genome sequencing (Additional file 6: Table S1). Stool specimens from patients with moderate AGE (Vesikari score; 8–10) and low Ct values (≤ 23) were included in the study. 30% w/v stool suspensions were prepared with phosphate-buffered saline (PBS; pH 7.4) and clarified by centrifugation at 10,000*g* for 10 min. Viral RNA was isolated using Trizol LS Reagent (Life Technologies, Grand Island, NY, USA) and eluted with 50 µl nuclease free water and used for whole genome analysis by NGS.

### cDNA library construction and illumina MiSeq sequencing

cDNA library preparation and Illumina MiSeq sequencing were carried out as described earlier [17]. Briefly, a 200-bp fragment library ligated with bar-coded adapters was constructed for individual strains with a NEBNext® Ultra™ II RNA Library Prep Kit for Illumina® (New England Biolabs, Ipswich, MA). Purification of the cDNA library was carried out with Agencourt AMPure XP magnetic beads (Beckman Coulter, Brea, CA). The quality assessment of the purified cDNA libraries was performed using a 4150 TapeStation (Agilent Technologies). About 151 paired end reads were generated by nucleotide sequencing by using an Illumina MiSeq sequencer (Illumina, San Francisco, CA) and a MiSeq Reagent Kit v2 (Illumina). CLC Genomics Workbench v7.0.3 (CLC Bio, Tokyo, Japan) was used to analyse the data. Contigs that shared a percent nucleotide identity of 95% or less were assembled from the obtained sequence reads by de novo assembly. The complete genome sequence of GII.17 strain (NICED-BCH-11889) was additionally determined by 5'-RACE [18]. Near-full genome sequences were deposited in DDBJ under the following accession numbers: LC769681.1–LC769715.1.

### Genotyping

Classification of genotypes is based on complete amino acid sequences of VP1 whereas P-typing is based on nucleotide sequences of the RNA dependent polymerase gene of ORF1. The human calicivirus typing tool [https://calicivirustypingtool.cdc.gov/] and the Norovirus typing tool 2.0 [https://www.rivm.nl/mpf/typingtool/norovirus/] [19, 20] were used for genotyping.

### Phylogenetic analysis

Multiple sequence alignments and phylogenetic analysis and were performed using MEGA 11 (Molecular Evolutionary Genetic Analysis ver 11.0.13) software. For the construction of phylogenetic tree best fitting substitution model was selected from model selection parameter in MEGA software. Substitution model GTR + G + I was chosen on the basis of lowest BIC and AIC(c) score. Pairwise sequence alignment and nucleotide or protein sequence identity were determined by using LALIGN tool (https://www.ebi.ac.uk/Tools/psa/lalign/) [21].

### Detection of recombinant norovirus and analysis of recombination breakpoints

To identify break points in the genomes of the recombinant strains, the sequences were analysed using SimPlot 3.5.1 and Simplot +  + software [22, 23]. The analysis was conducted using standard parameters of the program with a window size of 200 nt and a step size of 20 nt.

### RNA secondary structure prediction

Conserved RNA secondary structures upstream of the sub genomic transcript region were predicted using RNAalifold web server and mFold (RNA Folding Form ver. 2.3) software within the UNAFold web server.

### Detection of adaptive evolution and amino acid logo analysis

The number of non-synonymous substitutions per non-synonymous site (dN) and synonymous substitutions per synonymous site (dS) as well as dN/dS ratio was calculated. To identify sites under selection pressure, we employed the maximum likelihood (ML) based Fixed Effects Likelihood method (FEL) method, Bayesian approach based Fast Unconstrained Bayesian Approximation method (FUBAR), and a combination of ML and counting approach based Single-Likelihood Ancestor Counting method(SLAC) in Datamonkey 2.0 webserver [24]. P value < 0.1 was considered significant in SLAC and FEL method while posterior probabilities of > 0.9 was considered significant in FUBAR method.

Informative amino acid substitution has been identified by multiple protein sequence alignment tool in MEGA v11.0.13. The relative frequencies of amino acid occurrence (bits) of the proteins were visualized using WebLogo (ver. 3.7.11).

## Results

**Fig. 1:**
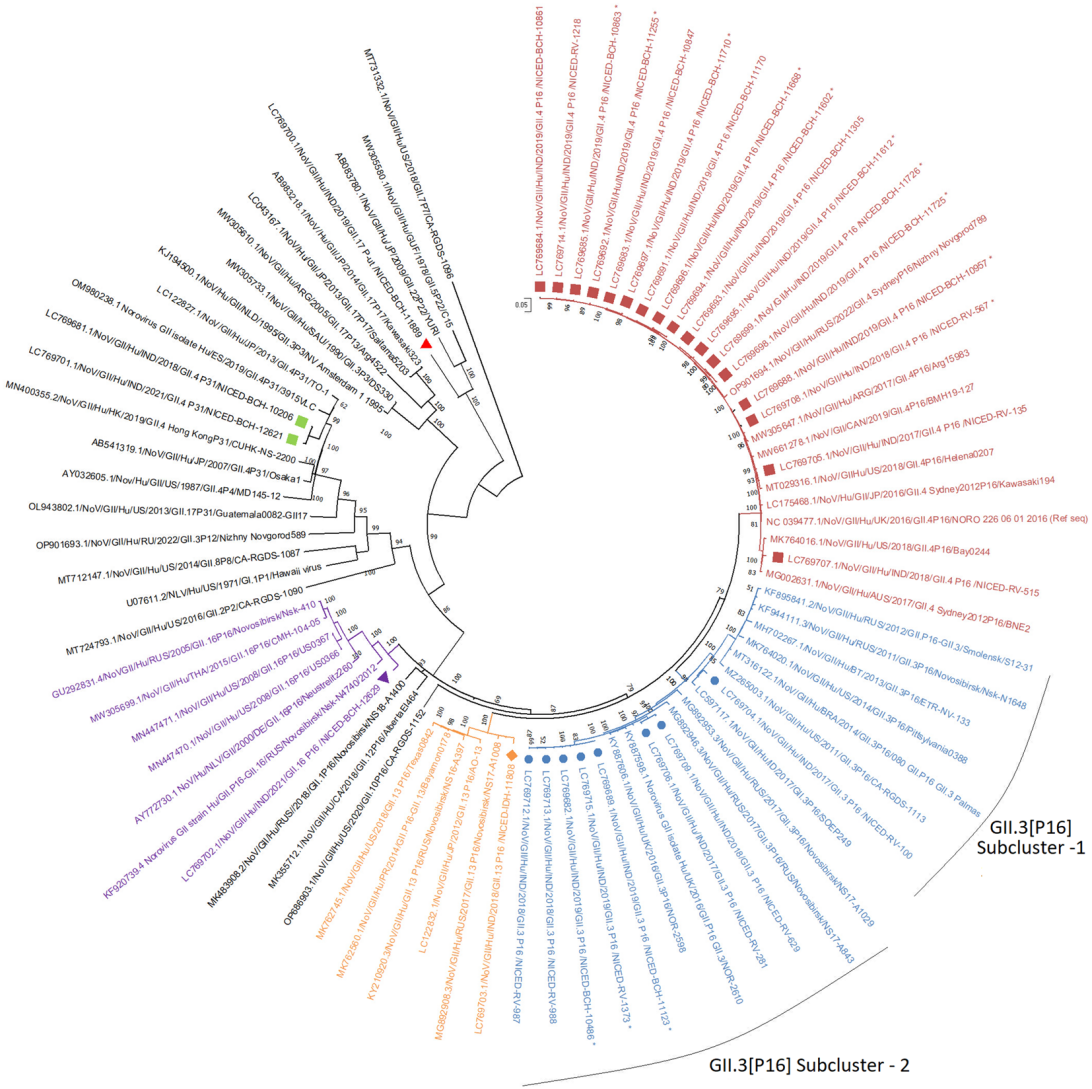
Maximum likelihood phylogenetic analysis of full genome sequences obtained from circulating GII noroviruses in eastern India. GII.4[P16] strains are marked in maroon square, GII.3[P16] are marked in sky blue circle, GII.13[P16] are marked in orange diamond, GII.16[P16] are in blue triangle, GII.4[P31] are in green square and GII.17[P-ut] are shown in red triangle. Bootstrap values < 70% are not shown. (*Partial sequences of genomic regions of noroviruses were reported previously and available in the Genbank database)

**Fig. 2:**
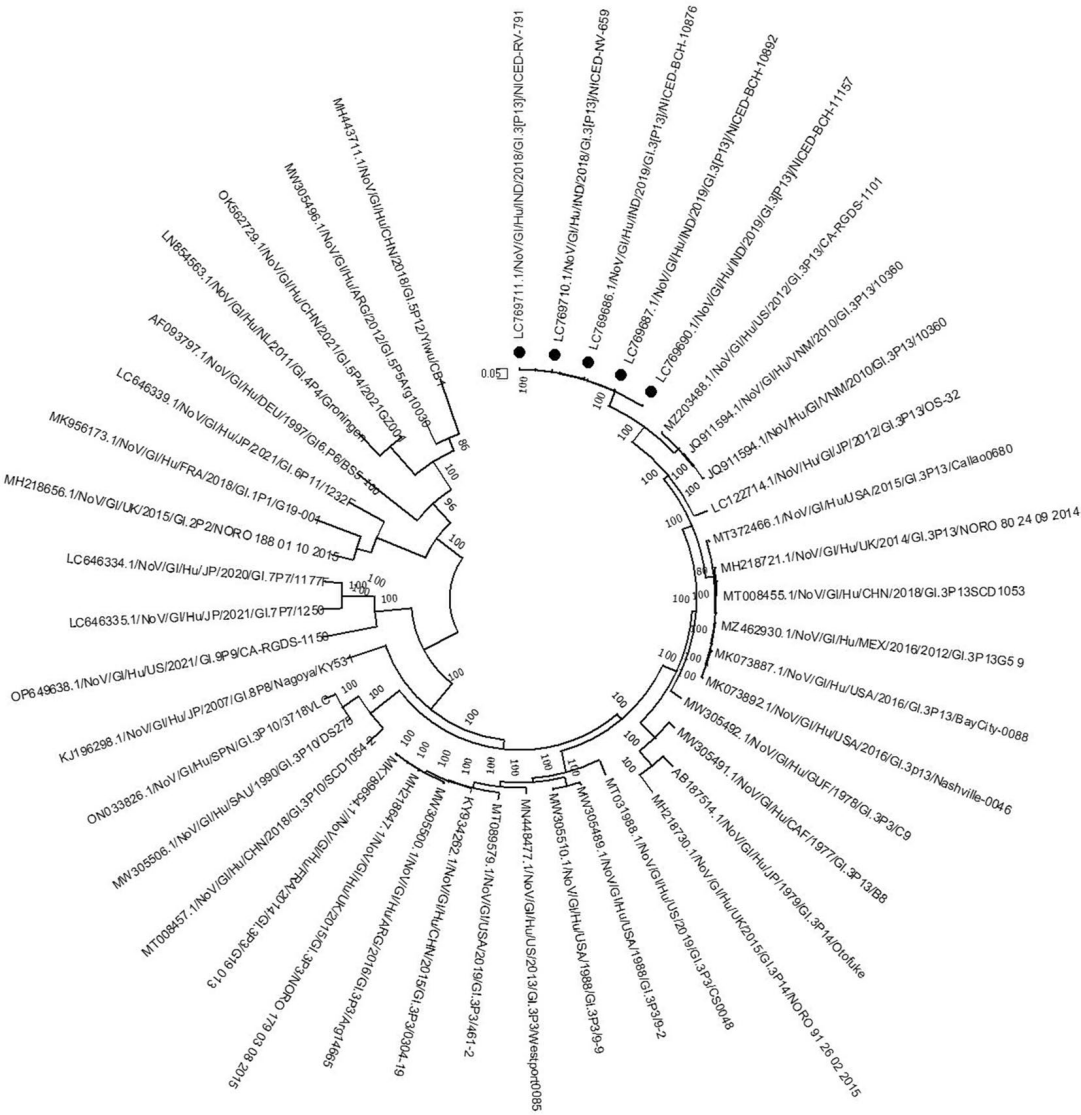
Maximum likelihood phylogenetic analysis of nearly complete genome sequences obtained from circulating GI noroviruses in eastern India. GI.3[P13] strains are marked in black circle. Bootstrap values < 70% are not shown

Of the 45 archived samples collected during October 2017 to July 2021, 35 (77.8%) resulted in near full genome sequences including 6 different capsid—polymerase (G-P types) combinations among GII genogroup. GII.4 Sydney [P16] was the commonest (n = 17) followed by GII.3[P16] (n = 8), GII.4 Sydney [P31] (n = 2), GII.13[P16] (n = 1), GII.16[P16] (n = 1) and GII.17[P untypeable] (n = 1) (Fig. 1). Five GI.3[P13] genomes were also identified during this study (Fig. 2). Phylogenetic trees of individual ORF1 and ORF2 of nearly full genome sequences of GII and GI noroviruses were analysed for better clarity and attached as Additional file 1: Fig. S1 and Additional file 2: Fig. S2 respectively. For the remaining 10 samples, 5 were genotyped (three GII.4 and two GII.13) using partial norovirus sequences however variant could not be determined. The other 5 samples could not be genotyped. The whole genome sequencing conducted in this current study reaffirmed that GII.4 was the predominant strain and followed by GII.3 strains, consistent with our previous study [12]. Notably, our findings in this study revealed that the [P16] polymerase genotype was the predominant genotype, accounting for 77.1% (27 out of 35 strains) of cases in Kolkata during the years 2017 and 2021. Overall, among GI and GII viruses, the diarrheal illness severity score of the patients was similar (Vesikari score; 8 to 10) (Additional file 6: Table S1).

### GII.4 sydney[P16]

Phylogenetic analysis of GII.4 Sydney[P16] strains showed most strains clustered together sharing nucleotide sequence identity ranging from 96.2 to 100% (Fig. 1). Phylogenetically they were closely related (95.7–97.9% identity) to previously reported GII.4 Sydney[P16] strains from Russia (OP901694.1), Argentina (MW305647.1), Canada (MW661278.1), USA(MT029316.1), Japan (LC175468.1) and the NCBI reference sequence of GII.4 Sydney[P16] genotype (NC_039477). Strain NICED-RV-515 was found to be distantly related (95.4–96.5% identity) to other eastern Indian GII.4[P16] strains and closely associated in the phylogenetic tree with previously reported strains from Australia (MG002631.1) and USA (MK764016.1) and shared 97.9–98% nucleotide sequence identity with them.

### GII.3[P16]

The GII.3[P16] strains formed two different sub-clusters; sub-cluster 1 and sub-cluster 2, with major strains belonging to sub-cluster 2 (Fig. 1). These strains shared 97.5–99.5% nucleotide sequence identity and are closely related (98.8–99.1% nucleotide sequence identity) with previously reported GII.3[P16] strains from the UK (NOR-2610/KY887598.1 & NOR-2598/KY887606.1). Two GII.3[P16] strains NICED-RV-629 and NICED-RV-281 clustered together with > 99% identity. One GII.3[P16] strain NICED-RV-100 clustered distantly in sub-cluster 1 and shared 97.4% sequence identity with the GII.3[P16] strain from Indonesia (S0EP249/LC597117.1). NICED-RV-100 strain shared 94.2% and 94.4% sequence identity with NICED-RV-281 and NICED-RV-629 strains in sub-cluster 2 respectively. The other eastern Indian GII.3[P16] strains with 93.7–94.1% nucleotide identity are distantly related to NICED-RV-100. Further analysis of ORF 2 region of NICED-RV-100 revealed only about 85.5–86.4% nucleotide sequence identity with ORF 2 region of GII.3 strains in sub-cluster 2 including other eastern Indian strains, suggesting circulation of heterogeneous GII.3 strain in this region.

### GII.4 sydney[P31]

GII.4[P31] strains NICED-BCH-10206 and NICED-BCH-12621 shared 98.9% nucleotide sequence identity among them and clustered with previously reported GII.4[P31] strains from Spain (OM980238.1) and Japan (LC122827.1) (Fig. 1).

Strain NICED-IDH-11808 clustered with GII.13[P16] inter-genotypic recombinant strains (about 94.8–97.3% nucleotide identity) from Russia (MG892908.3) and Japan (LC122832.1) during 2017 and 2012 respectively. NICED-BCH-12629 associated with GII.16[P16] strain from Russia (KF920739.4) sharing 93.7% nucleotide sequence identity. The amino acid sequences of NS7 (polymerase) region and phylogenetic analysis of ORF 1 of NICED-IDH-11808 (GII.13[P16]) and NICED-BCH-12629 (GII.16[P16]) revealed that these polymerases are the emerging GII[P16] polymerase type (Additional file 7: Table S2) [25].

### GI.3[P13]

Phylogenetic analysis of the eastern Indian GI.3[P13] strains revealed distinct clustering of these strains away from the previously reported GI.3[P13] strains. Only two GI.3[P13] strains MZ203488.1 and JQ911594.1 from USA and Vietnam shared more than 90% nucleotide identity with the eastern Indian GI strains (Fig. 2). The VP1 capsids of eastern Indian GI.3 noroviruses showed high nucleotide sequence identity (98.7–99.8%) among themselves, while closest nucleotide sequence identity (⁓ 99%) was observed among previously reported GI.3 strains from Taiwan (TW3108/MN922742.1 and TW3106/MN922741.1).

### Detection of recombination breakpoints

**Fig. 3:**
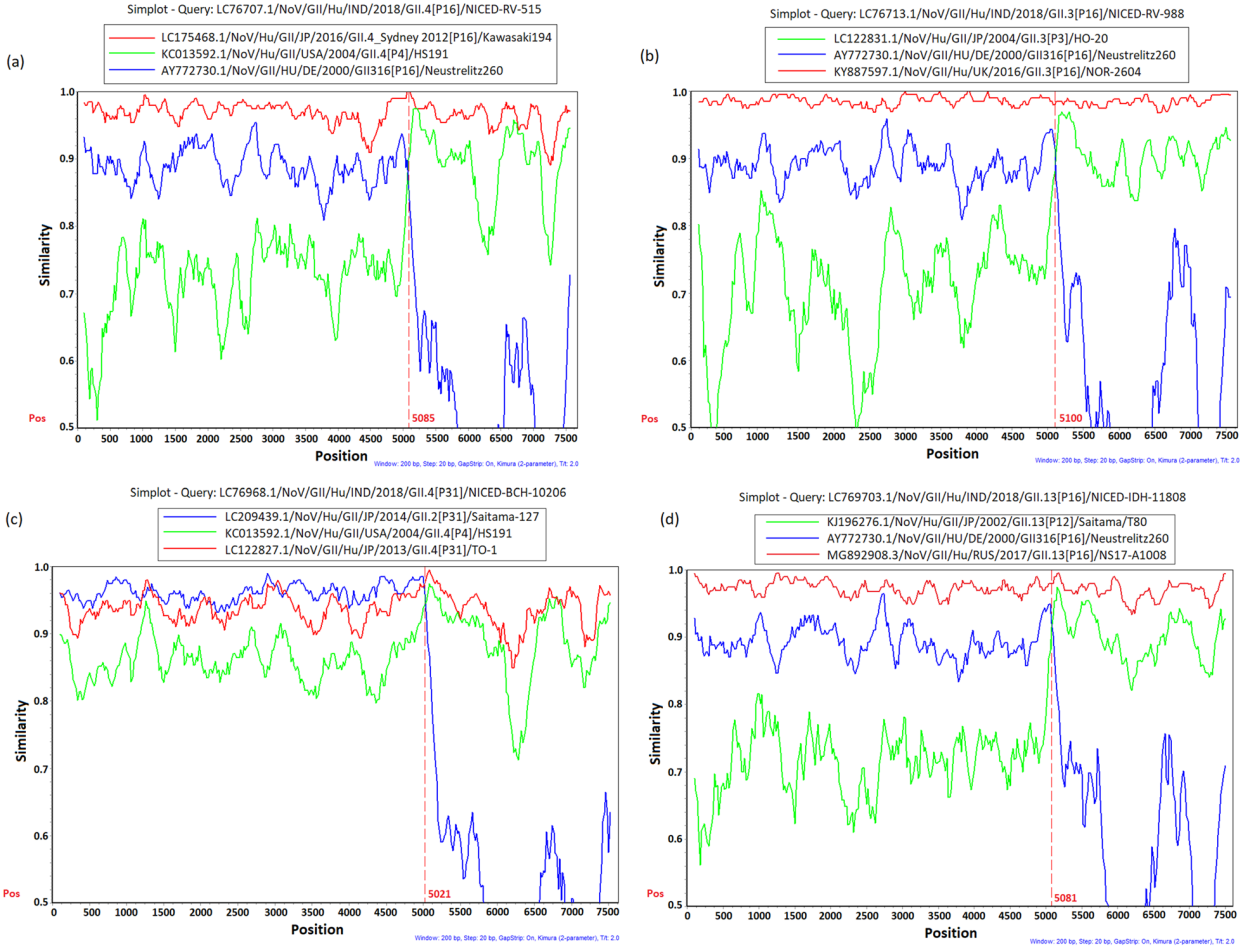
Simplot analysis of eastern Indian recombinant noroviruses. **a** SimPlot analysis of GII.4 Sydney[P16] strains were performed using reference parental strains GII.4[P4]/KC013592.1 (green line), GII.16[P16]/AY772730.1 (blue line) and reference recombinant strain GII.4[P16]/LC175468.1 (red line) was used as reference strain. **b** Simplot analysis of GII.3[P16] strains were performed using reference parental strain GII.16[P16]/AY772730.1 (blue line), GII.3[P3]/LC122831.1 (green line) and reference recombinant strain GII.3[P16]/KY887597.1 (red line). **c** SimPlot analysis of GII.4 Sydney[P31] strains performed using GII.4[P4]/KC013592.1 (green line) as reference parental strains for GII.4 and GII.2[P31]/LC209439.1 (blue line) was used as reference parental strains for GII[P31] genotype. GII.4[P31]/LC122827.1 (red line) was used as similar recombinant reference strain. **d** Simplot analysis of GII.13[P16] strain was performed using reference parental strain GII.16[P16]/AY772730.1 (blue line), GII.13[P12]/KJ196276.1 (green line) and reference recombinant strain GII.13[P16]/MG892908.1 (red line). Recombination breakpoints are marked in vertical red line (dashed)

To characterise the potential recombination events of the norovirus strains identified in this study, a similarity plot analysis was performed by aligning the complete genomic sequences of the study strains with closely related strains (Fig. 3). Similarity plot analysis of GII.4 Sydney[P16] strain NICED-RV-515 revealed recombination break point at approximately nt 5040, which is 32 nt upstream of the initiation site of ORF2 (nt position 5073; corresponding position to the nt 5085 of the reference strain GII.4[P16]/LC175468.1). In GII.3[P16] strains recombination breakpoint was detected approximately nt 5100 (corresponding nt position 5079 bp of reference strain GII.16[P16]/AY772730.1). In GII.13[P16] strain recombination breakpoint was observed approximately nt 5081 (corresponding nt position 5066 bp of reference strain GII.16[P16]/AY772730.1). Recombination breakpoints were also detected in GII.4 Sydney[P31] strains at approximately nt 5021 position (nt 5085 is the start site of ORF2) of reference strain GII.4[P4]/KC013592.1.

**Fig. 4:**
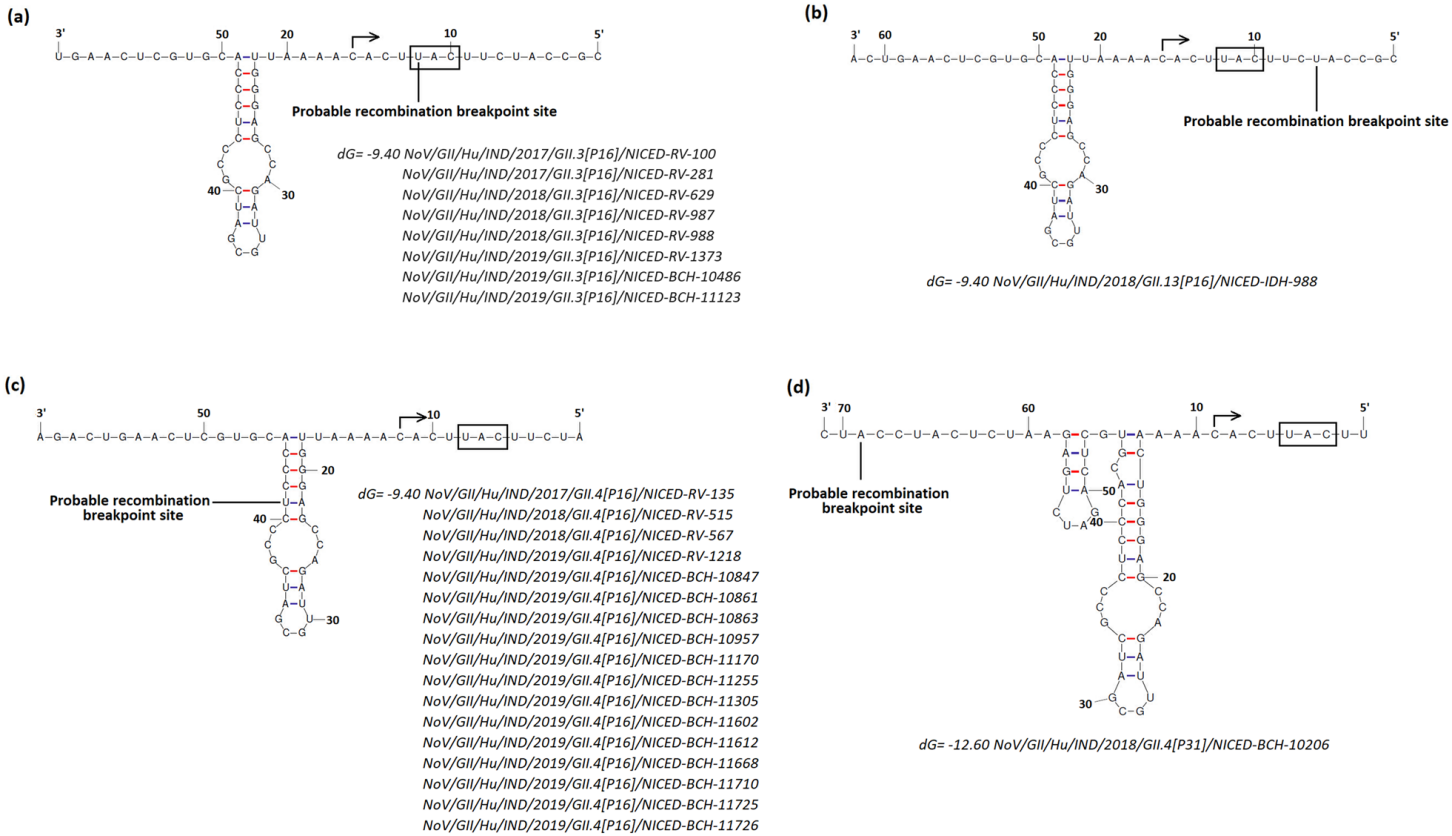
Analysis of sub-genomic transcription start region at ORF1/ORF2 junction of different norovirus strains circulated in eastern India; (**a**) GII.3[P16], (**b**) GII.13[P16], (**c**) GII.4 Sydney[P16] and (**d**) GII.4 Sydney[P31]. Predicted RNA secondary structure with least ΔG are shown in the diagram. For simplicity only one representative strain is shown in the diagram and other strains showing similar structure are listed below the diagram. The predicted secondary structures are shown in antisense orientation, sub-genomic RNA initiation sites are indicated by an arrow and the start anticodons are indicated in black box

Recombination breakpoints were analysed in the putative sub genomic transcription start region at NS/S junction (Fig. 4). In all GII.3[P16] and GII.13[P16] strains, recombination breakpoint was detected before or after the ORF2 start codon respectively (Fig. 4a, b). ln GII.4 Sydney[P16] and GII.4 Sydney[P31] strains probable recombination breakpoint was detected before ORF2 start codon but far away from sub genomic transcription start site (Fig. 4c, d). Interestingly, in all GII.4 Sydney[P16] strains probable recombination breakpoint was detected within the predicted secondary hairpin structure before the sub genomic transcription start region [26, 27].

### Analysis of predominantly circulating norovirus strains with GII.4 capsids

**Fig. 5:**
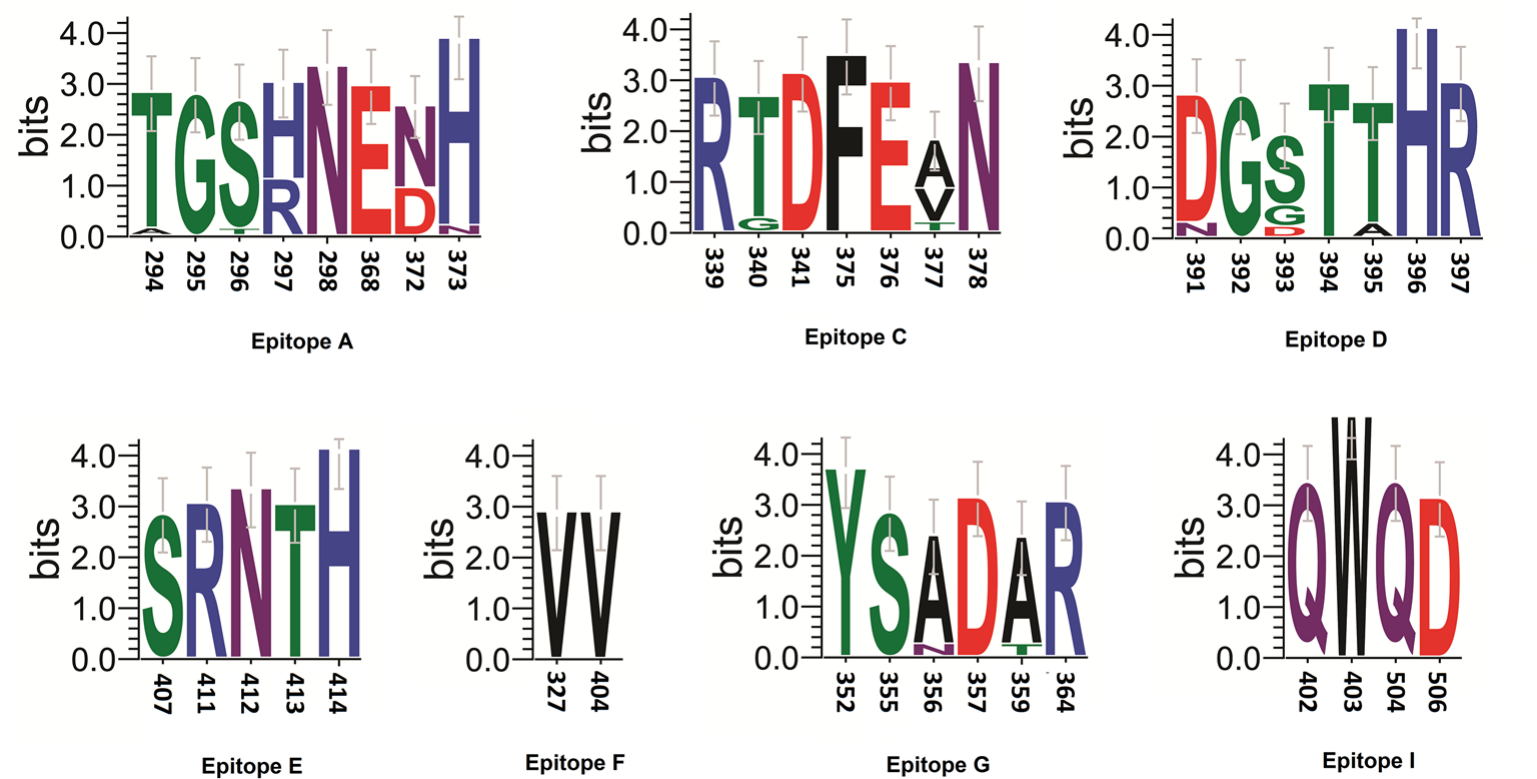
Analysis of variation in amino acid sequence of antigenic epitope regions of VP1 capsid among GII.4 Sydney strains detected in Kolkata, 2017–2021

Alignment of GII.4 capsid protein sequences showed variations in the antigenic epitopes (A, C, D, E, F, G and I) in the P2 subdomain. Amino acid residues in the Epitope E, F and I were the most conserved, while variation in amino acid residues were detected in other antigenic epitopes (Fig. 5 and Additional file 8: Table S3) [28]. Further analysis revealed alteration of amino acid sequences in the HBGA binding loops in the circulating GII.4 norovirus capsids (Additional file 9: Table S4).

### Detection of adaptive evolution in circulating GII[P16]

**Fig. 6:**
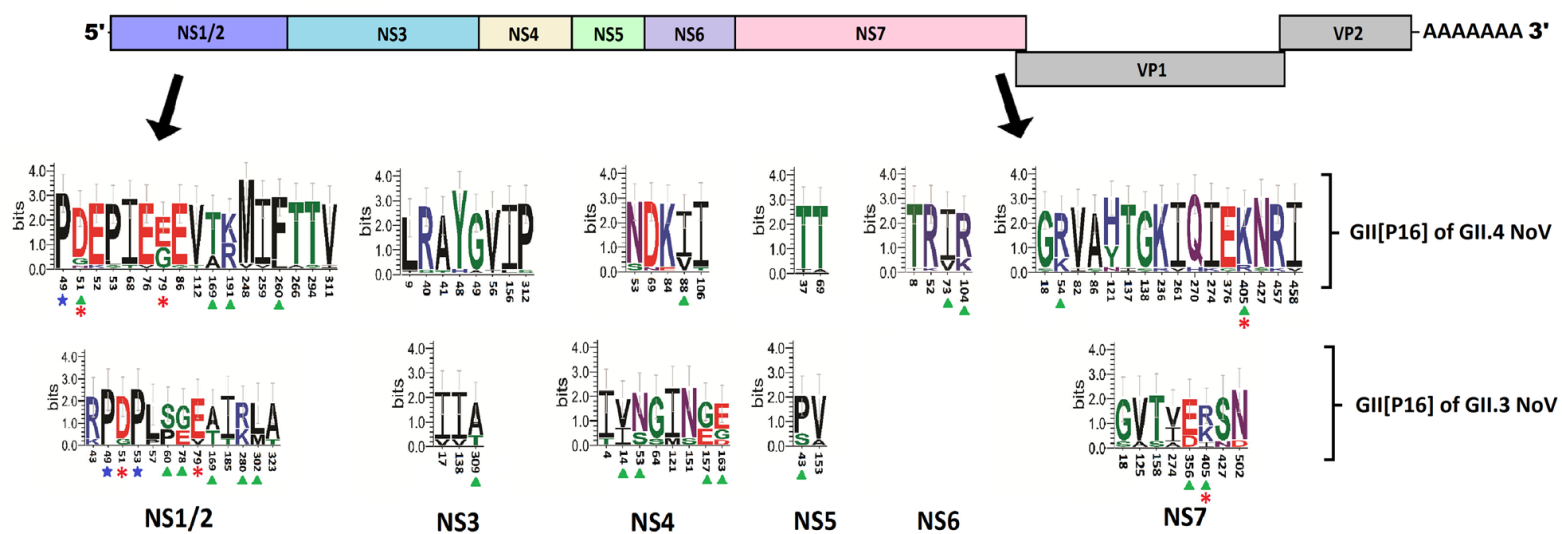
Analysis of informative amino acid substitutions in non-structural proteins of GII[P16] genotype associated with circulating GII.4 and GII.3 noroviruses. Amino acid substitution in circulating GII[P16] strains were compared with reference GII[P16] strain (NC_039477.1). Amino acid substitution with more than 5% frequencies were marked by green triangle and complete substitution sites were marked by blue star. Pervasive positive selection sites were indicated in red asterisk. (Details of this analysis are attached as Additional file 3: Fig. S3a–f)

The newly emerged GII[P16] polymerase types have acquired characteristics to combine with different capsid genotypes resulting in enhanced spread of this genotype globally. Analysis of amino acid sequences revealed several amino acid substitutions in the circulating ORF1 of GII.3[P16] and GII.4[P16] norovirus when compared to the reference GII[P16] strain (NC_039477.1) (Fig. 5). Several amino acid substitutions in the GII[P16] ORF1 of GII.3 viruses were different compared to GII[P16] ORF 1 of GII.4 viruses (Table 1 and Additional file 2: Fig. S3a–f). SLAC, FEL and FUBAR method analysis of the non-structural genes within the ORF1 of GII[P16] P-types revealed a large number of pervasive purifying selection sites (Additional file 4: Fig. S4). Two pervasive positive selection sites within the NS1/2 gene (NS1/2: D51G/N and NS1/2: E79G/V) and one in NS7 gene (NS7: K405R) were detected by only FUBAR method (marked by red asterisk in Fig. 6).

**Table 1:** Amino acid substitutions in the ORF 1 genes of GII[P16] P-types associated with GII.3 and GII.4 capsids compared to GII[P16] reference stain (NC_039477.1)

ORF1 genes	Substitutions in GII[P16] ORF1 of GII.3	Substitutions in GII[P16] ORF 1 of GII.4	Common substitutions in GII[P16] ORF1 of both GII.3 and GII.4
NS1/2 (p48)	R43K, S53P, P57L, P60S, E78G, E79V, I185T, K280R, L302M, A323T	D51N, E52K, S53L, I68T, E76V, E79G, E86G, V112T, R191K, M248V, I259V, F260L, T266A, T294S, V311I	S49P, D51G, T169A
NS3 (NTPase)	I17V, I138V, A309T	L9I, R40S, A41T, Y48H, G49A, V56I, I156L, P312S	
NS4 (p22)	I4T, I14V, G64S, I121M, N151S, E157G, E163G/D	D69N, K84E, V88I, I106T	N53S
NS5 (VPg)		T8I, R52K, I73V, K104R	
NS6 (Pro)	P43S, V153A	T37I, T69A	
NS7 (RdRp)	V125A, T158S, I274V, E356D, N502D	K54R, V82I, A86S, H121Y, T137I, G138S, K236R, I261V, Q270H, I274K, E376G, K457R, I458V	G18S, T158S, K405R, N427S

### Analysis of GII.17 norovirus

**Fig. 7:**
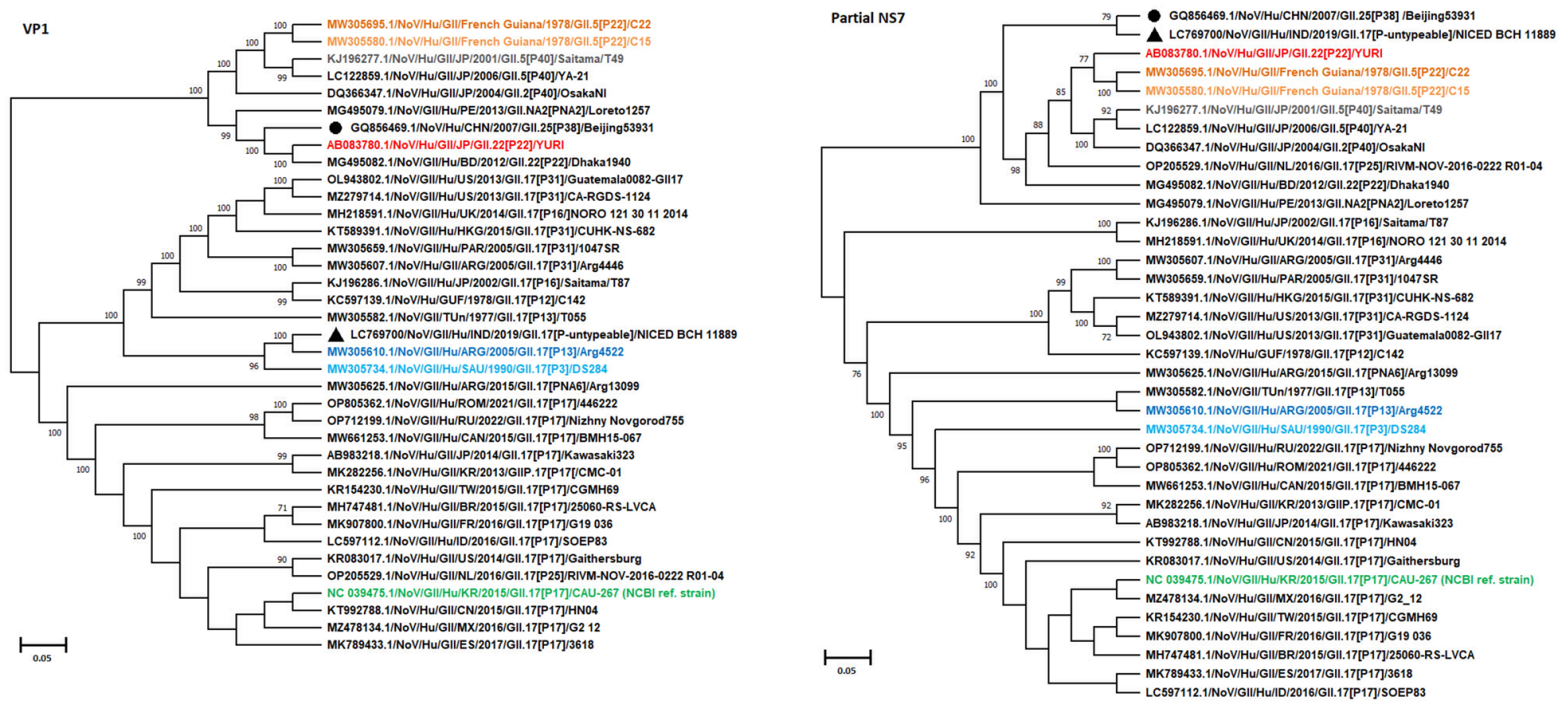
Phylogenetic analysis VP1 capsid and polymerase genes of a rare GII.17 norovirus strain NICED-BCH-11889. The closest similar polymerase strain Beijing53931/GII.25[P38] (GQ856469.1) was marked in black circle and the study strain was indicated in black triangle

In our previous study, among 90 norovirus positive specimens collected between January 2018 and December 2019, only one strain was identified as GII.17 based on the capsid gene [12]. In this study, we sequenced the entire genome of this specific GII.17 strain, designated as NICED-BCH-11889/2019. The capsid sequence of our GII.17 displayed a close phylogenetic relationship with two other strains: GII.17[P13] (MW305610/Argentina/2005) and GII.17[P3] (MW305734/Saudi Arabia/1990) strains, with bootstrap values ranging from 99 to 100% (Fig. 7). However, it is worth noting that the polymerase sequence of our GII.17 strain formed a distinct cluster, differing from the [P38] cluster, which includes two GII.25[P38] strains (GQ856469.1/China/2007 and HM635128.1/South Korea/2009) (Additional file 5: Fig. S5). The nucleotide identity between VP1 genes of NICED-BCH-11889/2019 and MW305610.1/Argentina/2005 was found to be 92.56%, indicating they share same GII.17 genotype. In contrast, the RdRp gene of NICED-BCH-11889/2019 exhibited a lower identity, ranging from 87.59 to 87.87% when compared to that of two GII.25[P38] strains.

**Fig. 8:**
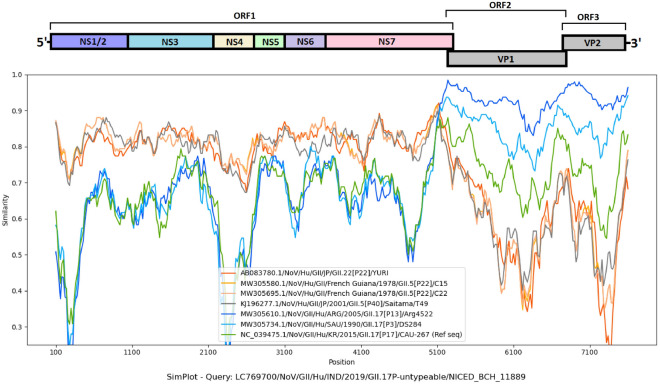
Sequence similarity of GII.17 NICED-BCH-11889 strain. The horizontal axis represents the nucleotide positions and the vertical axis represents the sequence similarity compared to the GII.17 NICED-BCH-11889 (LC769700.1) strain. The relative position of different ORFs of norovirus and polymerase-capsid junction was shown above the similarity plot

Similarity plot analysis revealed similarity between seven full genome sequences from NCBI and GII.17 norovirus NICED-BCH-11889 (Fig. 8). In the non-structural (ORF 1) region, YURI/GII.22[P22] (AB083780.1), C15/GII.5[P22] (MW305580.1), C22/GII.5[P22] (MW305695.1) and T49/GII.5[P40] (KJ196277.1) shared consistent similarity with GII.17 NICED-BCH-11889 strain. While in the capsid region (ORF2 and ORF3) a typical ‘W’ shaped pattern was observed in almost all strains, suggesting higher degree of variation in the hypervariable regions of capsid genes [29].

**Fig. 9:**
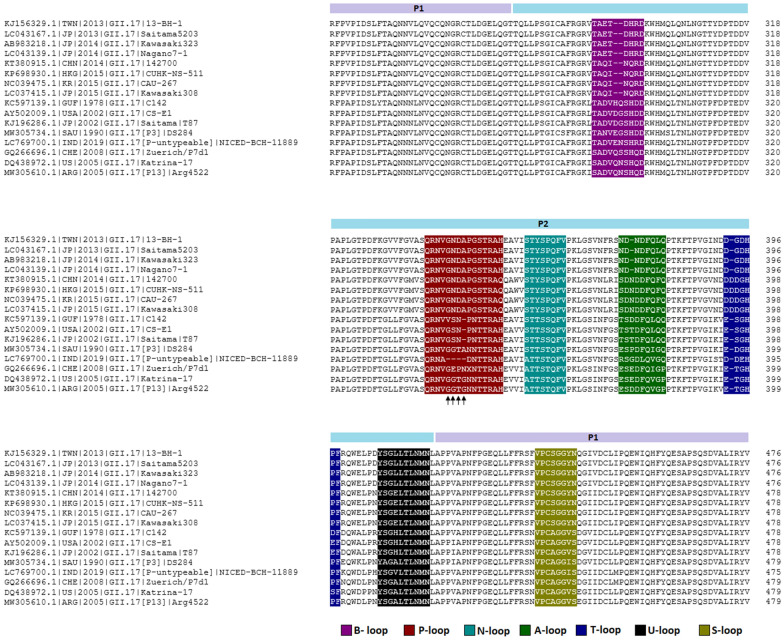
Amino acid sequence alignment of P domain from different GII.17 clusters. HBGA surface binding loops (A-, B-, P-, S-, T-, U- and N-) are heighted by different colours. The consecutive four amino acid deletions in the P- loop are marked by black arrows

Analysis of the histo-blood group antigen (HBGA) surface binding loops (A-, B-, P-, S-, T-, U- and N-) in the P- domain of VP1 capsid protein of NICED-BCH-11889 has identified novel changes in amino acid residues in the A-, B-, P- and T-loops compared to previously reported distinct GII.17 strains. Interestingly, a deletion of consecutive quadruple amino acid residues (aa343–aa346) in the P-loop of NICED-BCH-11889 strain was also noteworthy (Fig. 9).

## Discussion

Genomic plasticity leading to emergence of diverse recombinant strains and limited cross-protection against viruses from different genotypes have made studies better understanding their evolution important. Although whole genome noroviruses from many different countries are available, only one such report is available from India [30]. Our study provides a comprehensive analysis of norovirus among patients with acute diarrheal illness in eastern India. In contrast to other studies, where increased severity of diarrheal illness is associated with GII.4 viruses compared to non GII.4 viruses, no conclusive correlation between disease severity and individual genotypes was observed in our study [31–33]. The limitation of our study is the relatively low number of samples (n = 45) that were studied.

Genetic diversity of noroviruses is shaped by evolution and recombination events primarily among viruses within the same genogroup [34]. To date, of the four reported inter-genogroup recombinant norovirus strains, one strain (polymerase sequence of genogroup GI and capsid sequence of genogroup GII and genotype 4 (GII.4)) was detected from Kolkata, India in 2006 [35, 36]. In the present study, all of the recombinants identified were inter-genotypic in nature. Most recombination events take place at the ORF1/ORF2 junction region leading to multiple combinations of capsid and polymerases, generating new recombinant strains that might have advancement in fitness, pathogenesis and/or transmissibility compared to their ancestral strains [37, 38]. Changes in the VP1 enable norovirus to evade immunity developed against prior infection which has been proposed as the mechanism of changing GII.4 variant viruses between 2002 and 2015. [5]. However, in recent years several genotypes have a novel ORF1 and polymerase types (e.g. GII.P16) [25]. Emergence of a new GII[P16] ORF1 associated with multiple capsid genotypes (e.g., GII.4 Sydney[P16], GII.2[P16], GII.3[P16], GII.12[P16]) has been reported worldwide [39] and were also found in our study. The GIIP[16] polymerases detected in our study harboured many mutations in the part of ORF1 encoding non-structural proteins (NS1/2, NS3-NS6) other than the polymerase. Some of these mutations (like amino acid residue no. 52, 53 and 76 of NS1/2) were similar to those reported in other GII[P16] polymerases from around the world [25].

Since 2002, new GII.4 variants replacing previous dominant GII.4 variants have emerged every 2–3 year until GII.4 Sydney emerged in 2012 and this GII.4 variant is till dominant in 2023. Out of the 5 antigenic epitope regions mapped on the capsid protein of the circulating GII.4 viruses, Epitope A is the most dynamic, conferring immune-elusive property to the virus. Though more similar in amino acid composition to the GII.4 Sydney 2012 capsid, heterogeneity among endemically circulating strains was evident at positions 297 and 372 of epitope A. Residues 297 and 372 residing close to each other on the capsid region have been found to be co-evolutionary in generating antigenic variants of GII.4 capsids [40]. Seven of our GII.4 strains had histidine and asparagine at positions 297 and 372 similar to GII.4 Sydney 2012 while other GII.4 capsids such as Grimsby 1995 and Farmington Hills 2002 GII.4 variants retained arginine and aspartic acid at these positions. Among the GII.4 Sydney [P16] strains, some had valine substituted for alanine at position 377 which was unique to the strains in our study. The GII.4 Sydney capsids with [P31] polymerases differed from most of the GII.4 strains (endemic GII[P16] and established antigenic variants of GII.4) at residue 340 of epitope C (glycine in place of threonine), residue 393 (aspartic acid instead of alanine/serine) and 395 (threonine in place of alanine) of epitope D and residue 356 (asparagine in place of alanine) and 359 (threonine in place of alanine) at epitope G. These substitutions characterised the GII.4 capsids of both the [P31] polymerases detected in this study. The norovirus vaccine TAK-214 is a bivalent vaccine comprised of GI.1 and GII.4c component. The GII.4c component is a consensus sequence of three different GII.4 genotype variants—Yerseke_2006a(ABL74391.1), Den Haag_2006b(ABL74395.1) and Houston_2002 (ABY27560.1) (Additional file 8: Table S3) [41–43]. Compared to the GII.4c construct included in the bivalent TAK-214 norovirus vaccine, the east-Indian GII.4 capsids had 6–7 amino acid differences in epitope A, 2 in epitope D and 5 in epitope G. Further serology studies are necessary to understand the degree of immunogenicity that may be conferred by this vaccine against the currently circulating norovirus strains.

Globally, many countries have reported various sporadic cases of non-GII.4 strains like GII.3, GII.13 and GII.17, all of which can form chimeric viruses with various P-types like [P4], [P13], [P16], [P25], and [P31] [39, 44–46]. Previous studies in eastern India have reported parental norovirus strains like GII.13[P13] and GII.16[P16], but in our study chimeric norovirus strains like GII.3[P16] and GII.13[P16] were detected besides GII.16[P16] parental strain, potentially suggesting co-circulation of both parental and recombinant strains [13]. Since 2015, GII.17 viruses have temporarily become predominant and were detected in multiple countries [47–51]. The newly emerged GII.17 was associated with the [P17]-type. However, the sporadic GII.17 strain (NICED-BCH-11889) in this study was genotyped as [Puntypeable]. Though the P-type of our GII.17 strain (NICED-BCH-11889) showed closest similarity with GII[P38] type, low nucleotide identity between the two suggests that this polymerase genotype is not GII[P38] P-type. This observation led us to classify our GII.17 strain as [P untypeable] for its polymerase gene, as determined by the human calicivirus typing tool. Although the capsid gene was also genotyped as GII.17, it exhibited a closer relationship to the VP1 genes of GII.17[P13] and GII.17[P3] norovirus strains. These findings suggest that our GII.17 norovirus strain may not have originated from the newly emerged GII.17[P17] strain, which was widespread and dominant in many countries during 2015–2017. Instead, our GII.17 strain may have originated from sporadic GII.17[P13] and GII.17[P3] strains, possibly undergoing further recombination or evolution in its RdRp genes.

Several distinct variants of GII.17 have evolved since 1976, with alterations in the surface exposed loops of the P2 subdomain of VP1 capsid protein [29, 52]. However, the GII.17 (NICED-BCH-11889) strain detected in our study is distinct from other GII.17 variants with mutations in A, B, P and T loops, particularly a four amino deletions in the P-loop on the protein's outer surface, that might have altered its binding ability with HBGA.

## Conclusion

We determined and analyzed 35 full genome norovirus sequences from stool specimens collected between October 2017 to July 2021 from hospitalized children in two hospitals in Kolkata, India. Comprehensive molecular analyses demonstrated that a large variety of different genotypes, similar to those that have been reported globally providing the start of baseline information on which strains a future norovirus vaccine should protect against to reduce the burden of moderate to severe norovirus gastroenteritis in eastern India.

### Supplementary Information

**Additional file 1: Figure S1.** Maximum likelihood phylogenetic analysis on the basis of nearly complete ORF1 and complete ORF2 region of circulating GII noroviruses in eastern India during 2017–2021.

**Additional file 2: Figure S2.** Maximum likelihood phylogenetic analysis on the basis of nearly complete ORF1 and complete ORF2 region of circulating GI noroviruses in eastern India during 2017–2021.

**Additional file 3: Figure S3a–f.** Alignment of amino acid sequences of ORF1 encoded genes [NS1/2(a), NS3(b), NS4(c), NS5(d), NS6(e) and NS7(f)] of GII.3[P16] and GII.4[P16] noroviruses.

**Additional file 4: Figure S4.** Number of pervasive purifying selection sites in non-structural proteins of commonly circulating GII[P16] strains by SLAC, FEL and FUBER method.

**Additional file 5: Figure S5.** Phylogenetic tree of GII.17 and other strains.

**Additional file 6: Table S1.** Details of clinical samples enrolled in this study.

**Additional file 7: Table S2.** Alignment of unique informative amino acid sites in the of ORF-1 non-structural encoded proteins (NS1/2, NS4, NS6 and NS7) of GII[P16] genotype (after Barclay et al., 2019). Informative amino acid sites in the pre-2015 GII[P16] are labelled in light red, novel GII[P16] are labelled in green and Indian GII[P16] strains are labelled in blue.

**Additional file 8: Table S3.** Alignment of antigenic epitope regions in the P2 subdomain of VP1 capsid protein of circulating GII.4 strains with vaccine strain GII.4c and different antigenic variants of GII.4 strains.

**Additional file 9: Table S4.** Analysis of amino acid sequences in the HBGA binding pockets of P domain in GII.4 capsid proteins. (Completely conserved or mostly conserved regions are highlighted in dark red and variable amino acid residues are highlighted in yellow).

## Data Availability

The data that support the findings of this study are available in DDBJ at https://www.ddbj.nig.ac.jp/index-e.html under the Accession numbers LC769681.1—LC769715.1, which is also available in NCBI database.
