# Supplementary figures and images for: Comprehensive full genome analysis of norovirus strains from eastern India, 2017–2021
Source: Gut Pathog. 2024 Jan 18;16:3. doi: 10.1186/s13099-023-00594-5 (PMC10797879; doi:10.1186/s13099-023-00594-5)

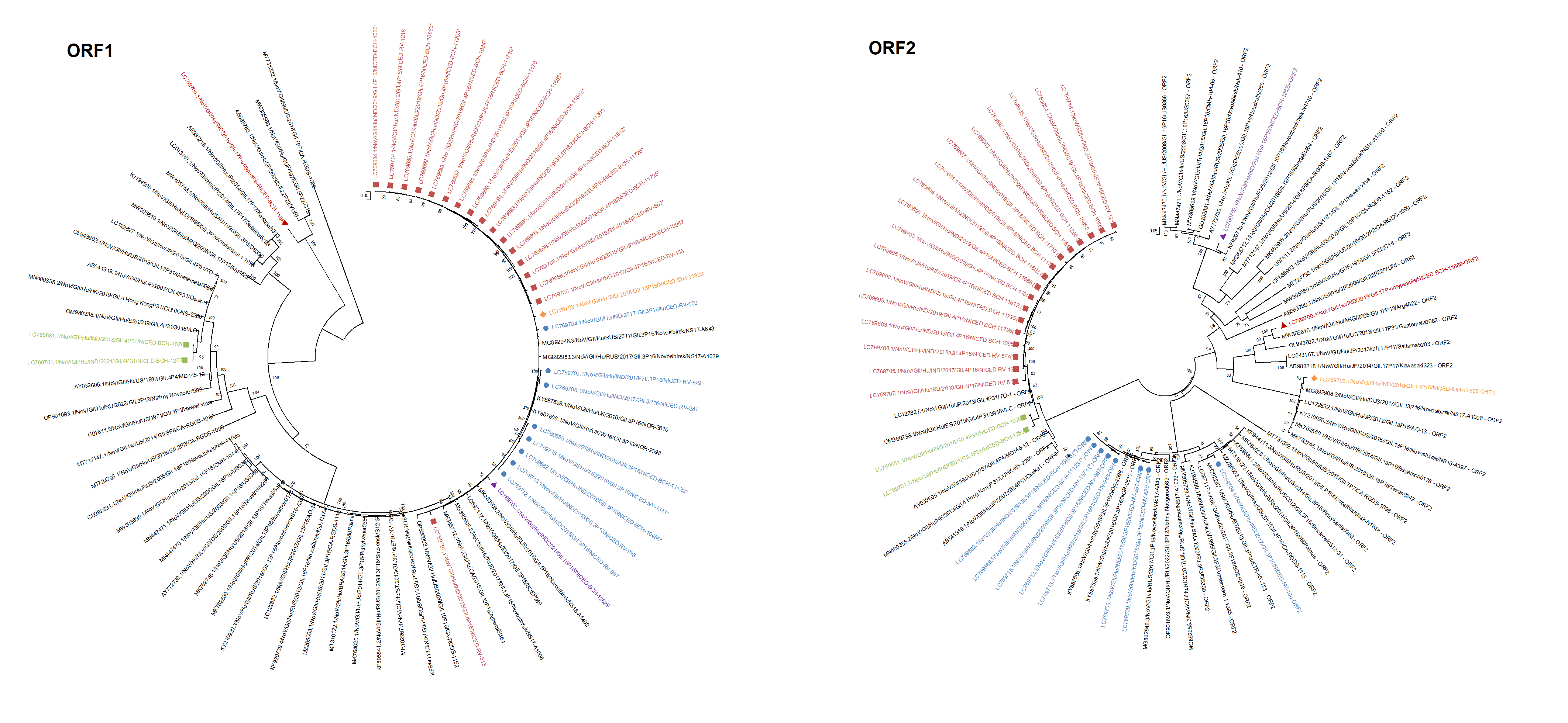

Supplement: Supplementary file 1 — Additional file 1: Figure S1. Maximum likelihood phylogenetic analysis on the basis of nearly complete ORF1 and complete ORF2 region of circulating GII noroviruses in eastern India during 2017–2021. [file 13099_2023_594_MOESM1_ESM.tif]

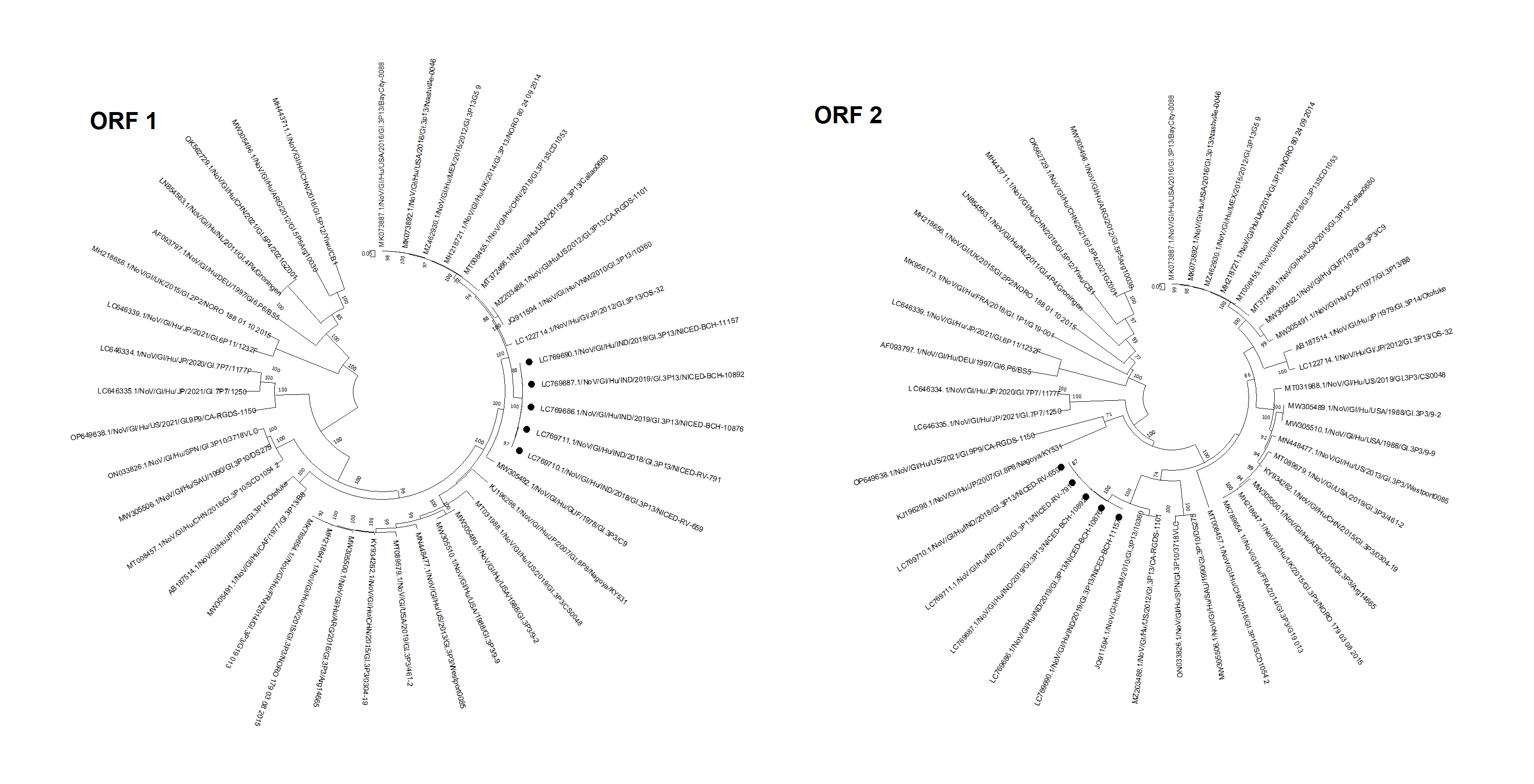

Supplement: Supplementary file 2 — Additional file 2: Figure S2. Maximum likelihood phylogenetic analysis on the basis of nearly complete ORF1 and complete ORF2 region of circulating GI noroviruses in eastern India during 2017–2021. [file 13099_2023_594_MOESM2_ESM.tif]

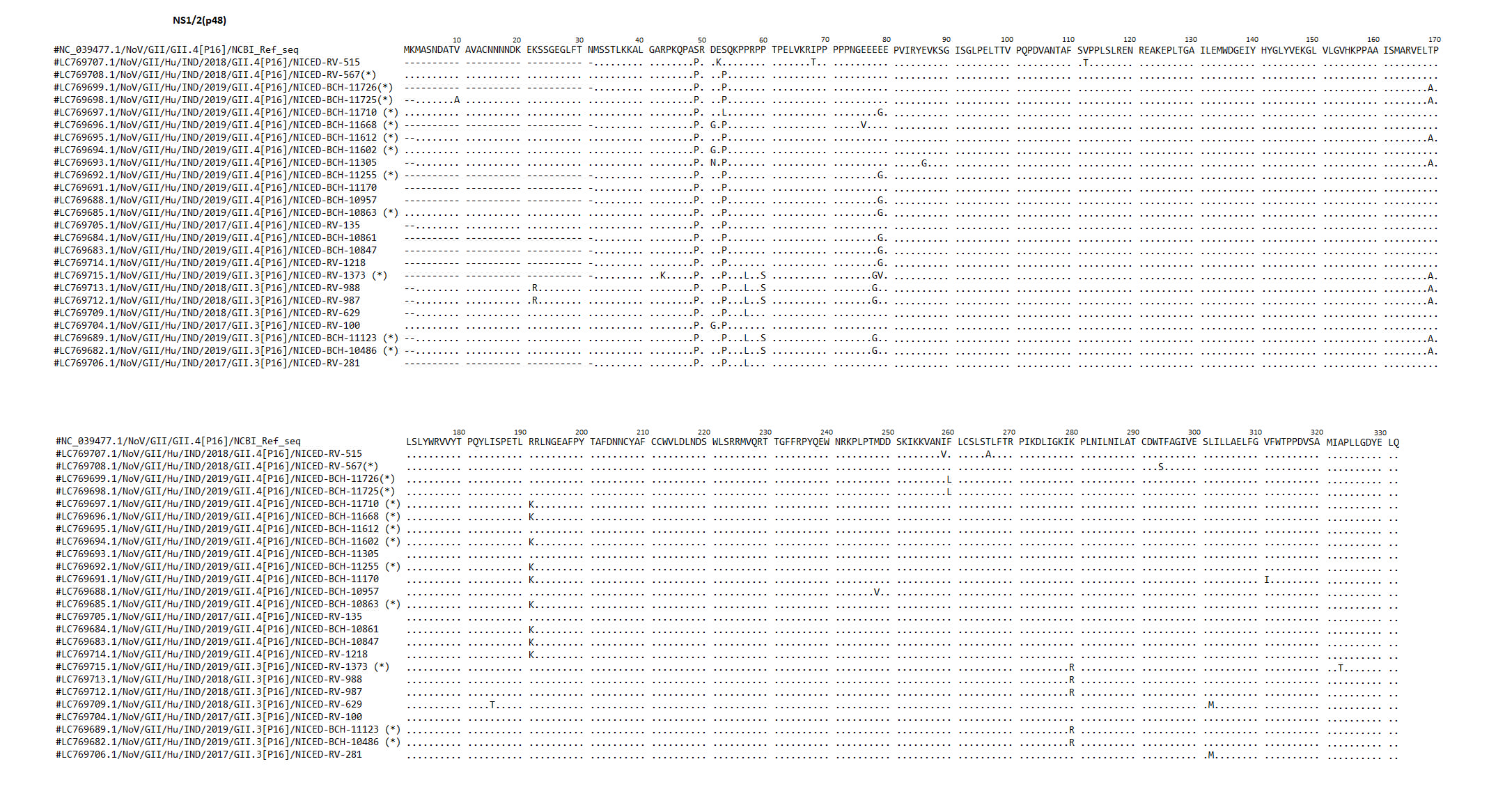

Supplement: Supplementary file 3 — Additional file 3: Figure S3a–f. Alignment of amino acid sequences of ORF1 encoded genes [NS1/2(a), NS3(b), NS4(c), NS5(d), NS6(e) and NS7(f)] of GII.3[P16] and GII.4[P16] noroviruses. [file 13099_2023_594_MOESM3_ESM.zip › New folder/Supplementary figure 3a.tif]

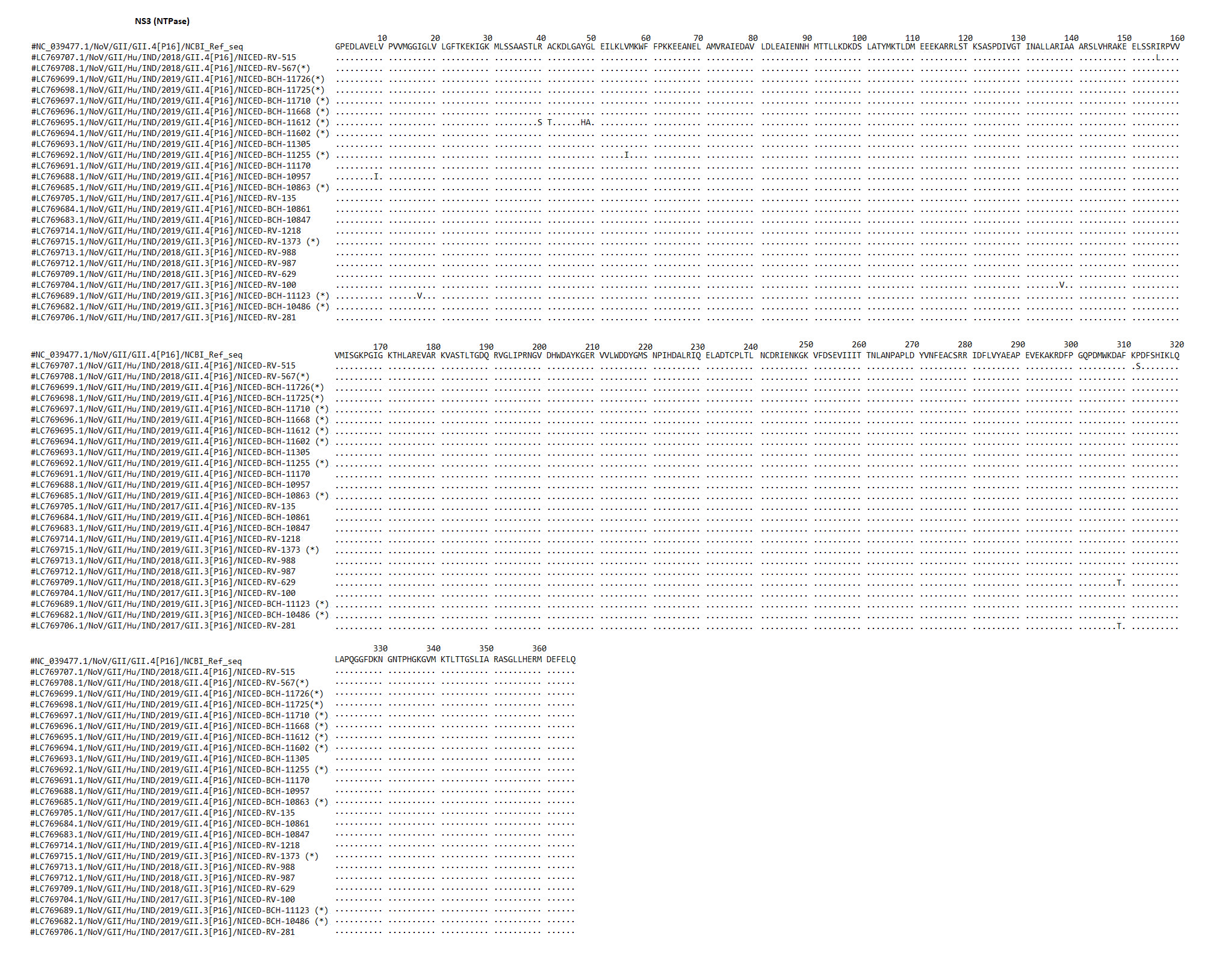

Supplement: Supplementary file 3 — Additional file 3: Figure S3a–f. Alignment of amino acid sequences of ORF1 encoded genes [NS1/2(a), NS3(b), NS4(c), NS5(d), NS6(e) and NS7(f)] of GII.3[P16] and GII.4[P16] noroviruses. [file 13099_2023_594_MOESM3_ESM.zip › New folder/Supplementary figure 3b.tif]

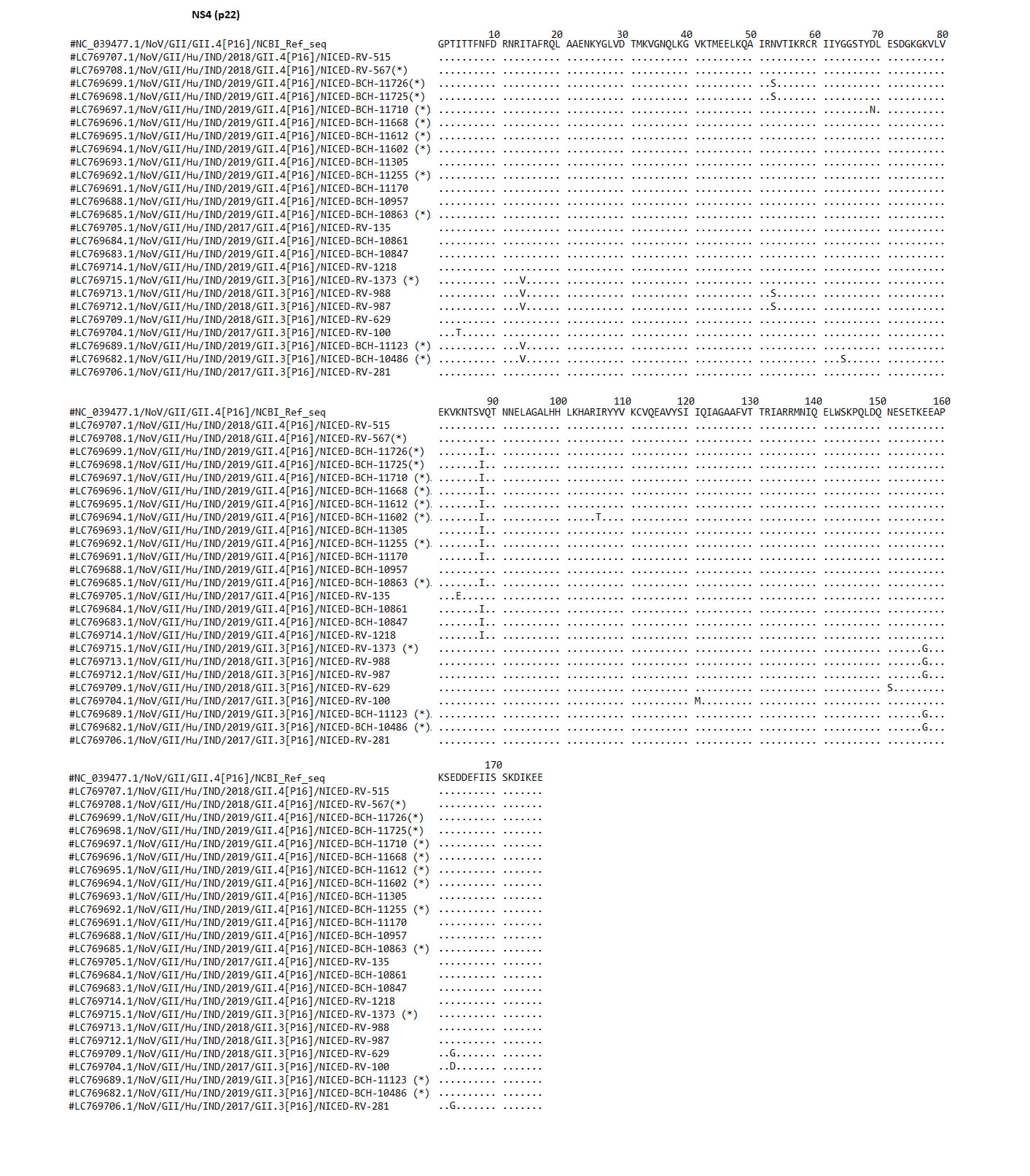

Supplement: Supplementary file 3 — Additional file 3: Figure S3a–f. Alignment of amino acid sequences of ORF1 encoded genes [NS1/2(a), NS3(b), NS4(c), NS5(d), NS6(e) and NS7(f)] of GII.3[P16] and GII.4[P16] noroviruses. [file 13099_2023_594_MOESM3_ESM.zip › New folder/Supplementary figure 3c.tif]

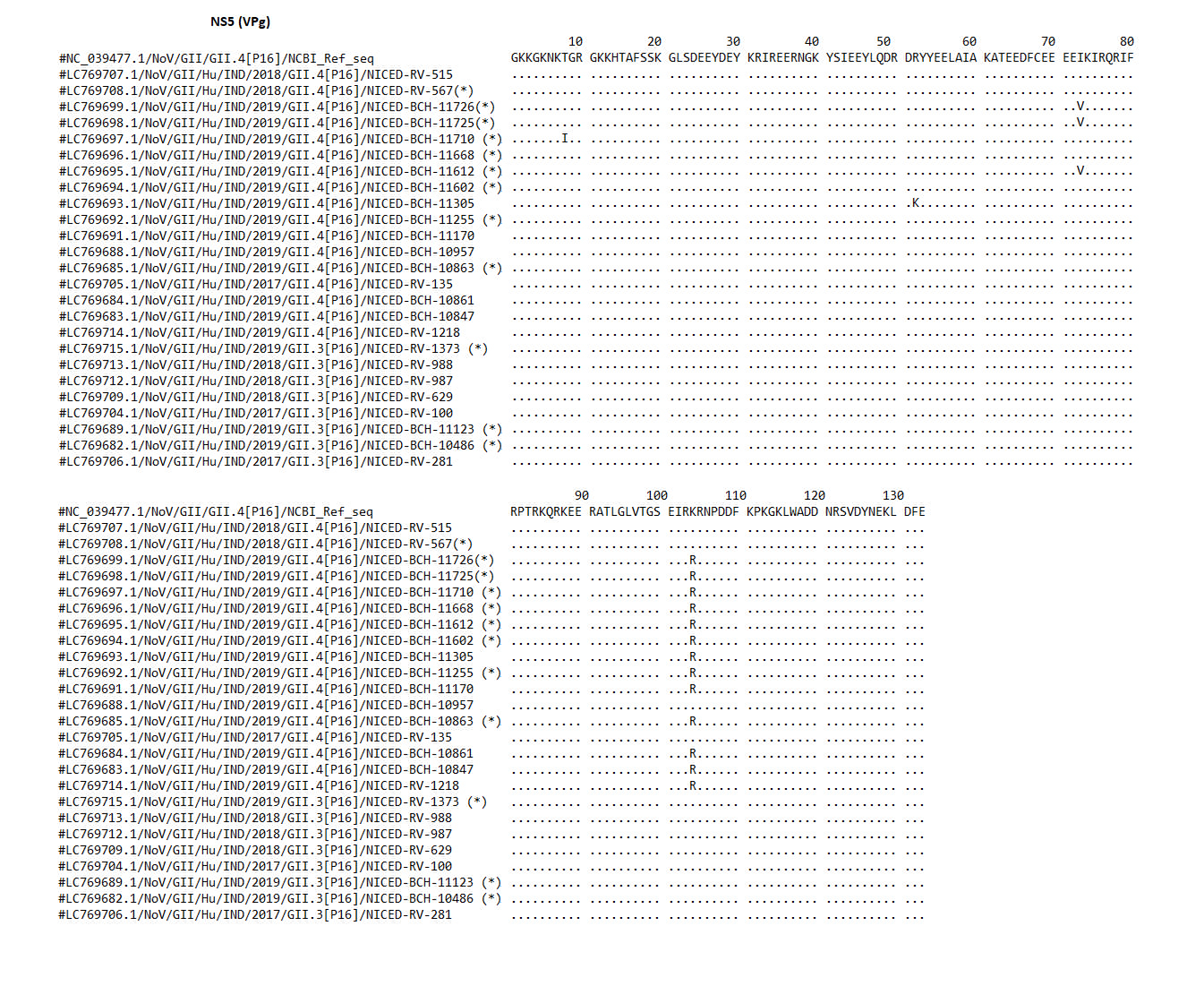

Supplement: Supplementary file 3 — Additional file 3: Figure S3a–f. Alignment of amino acid sequences of ORF1 encoded genes [NS1/2(a), NS3(b), NS4(c), NS5(d), NS6(e) and NS7(f)] of GII.3[P16] and GII.4[P16] noroviruses. [file 13099_2023_594_MOESM3_ESM.zip › New folder/Supplementary figure 3d.tif]

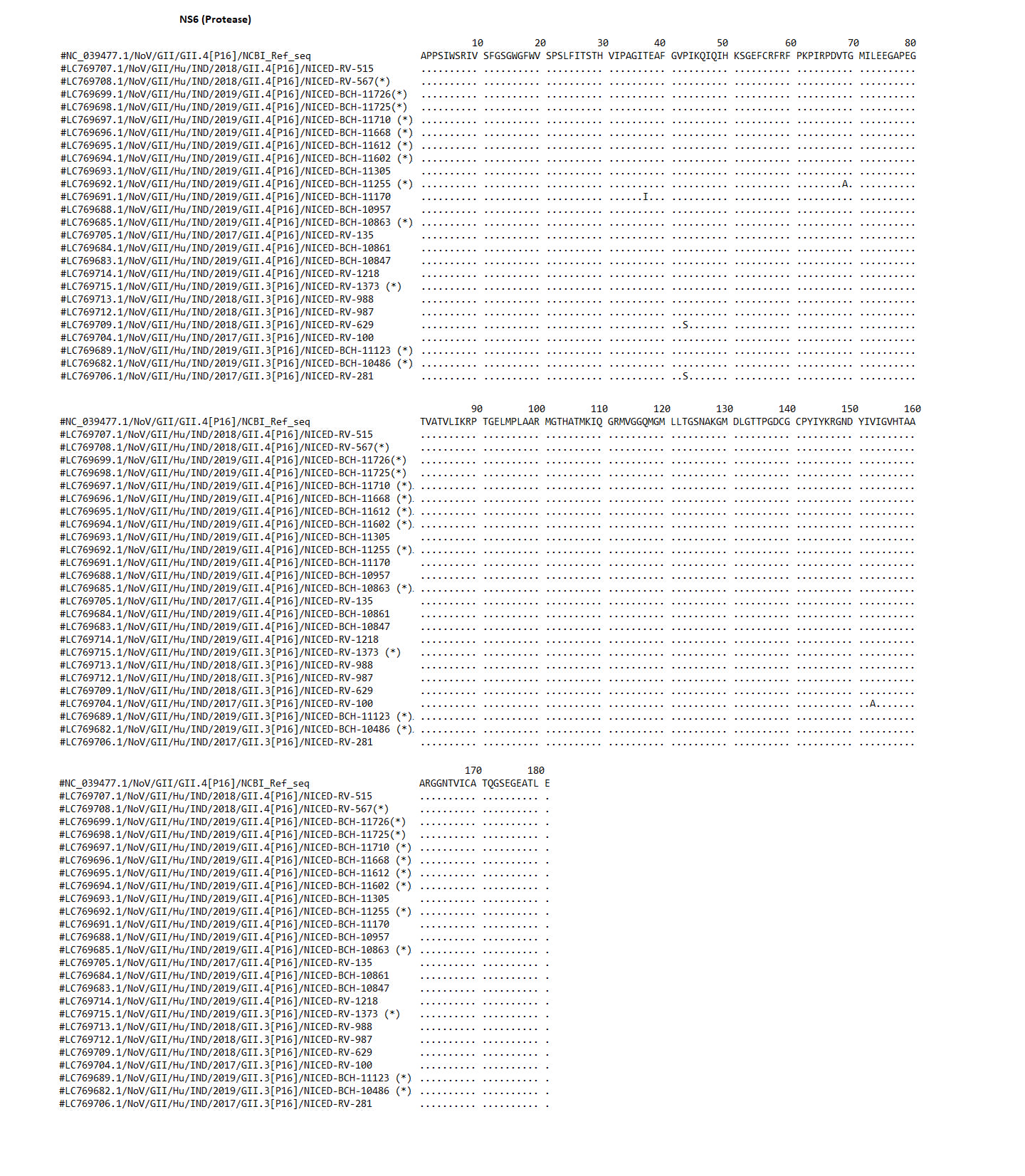

Supplement: Supplementary file 3 — Additional file 3: Figure S3a–f. Alignment of amino acid sequences of ORF1 encoded genes [NS1/2(a), NS3(b), NS4(c), NS5(d), NS6(e) and NS7(f)] of GII.3[P16] and GII.4[P16] noroviruses. [file 13099_2023_594_MOESM3_ESM.zip › New folder/Supplementary figure 3e.tif]

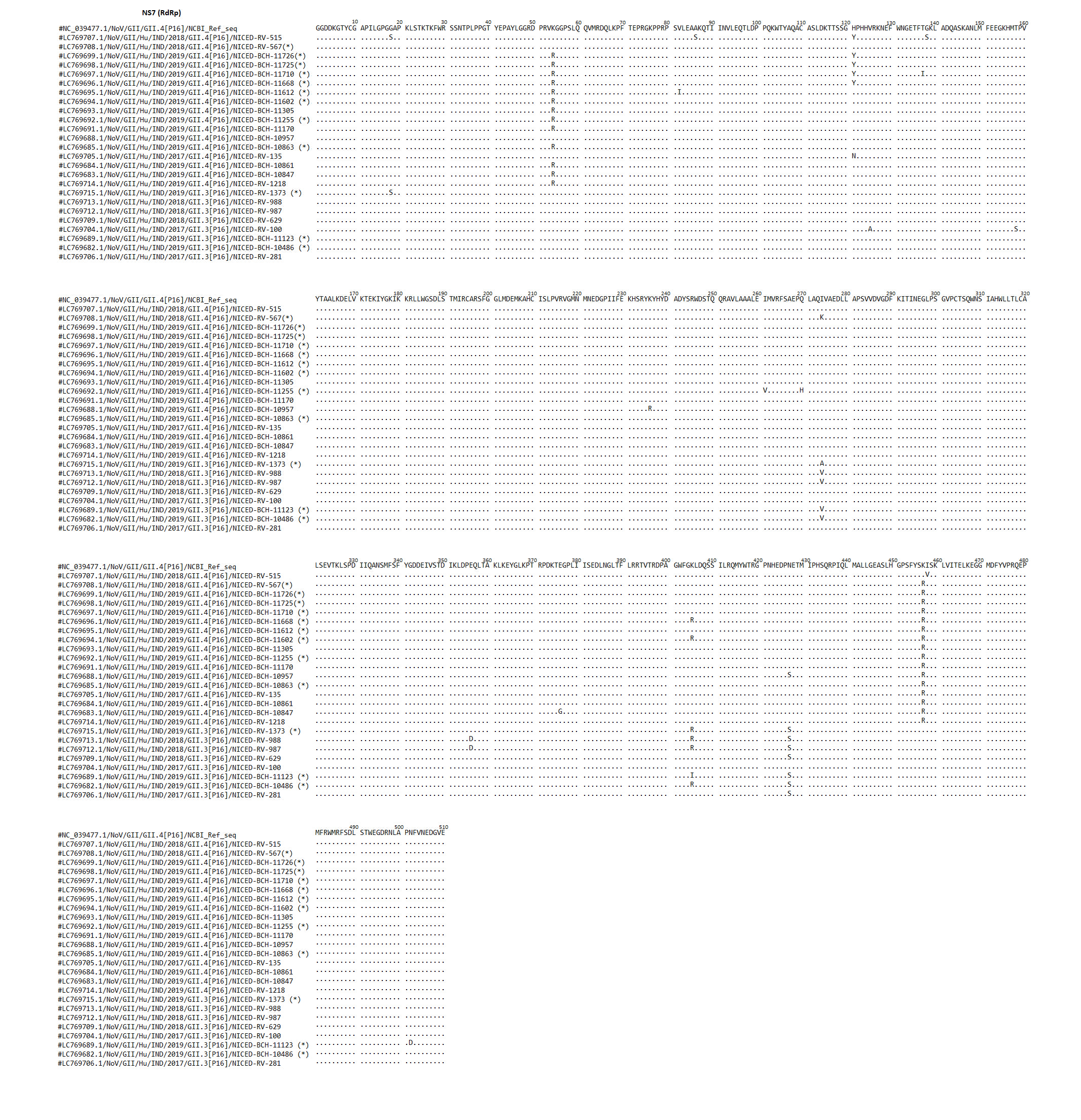

Supplement: Supplementary file 3 — Additional file 3: Figure S3a–f. Alignment of amino acid sequences of ORF1 encoded genes [NS1/2(a), NS3(b), NS4(c), NS5(d), NS6(e) and NS7(f)] of GII.3[P16] and GII.4[P16] noroviruses. [file 13099_2023_594_MOESM3_ESM.zip › New folder/Supplementary figure 3f.tif]

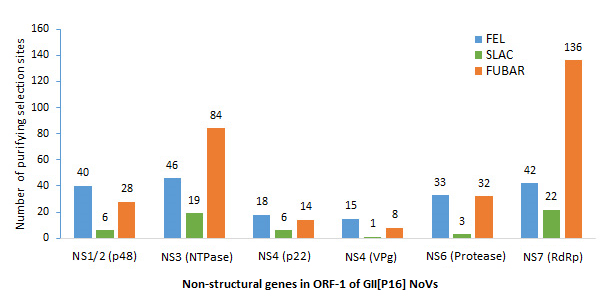

Supplement: Supplementary file 4 — Additional file 4: Figure S4. Number of pervasive purifying selection sites in non-structural proteins of commonly circulating GII[P16] strains by SLAC, FEL and FUBER method. [file 13099_2023_594_MOESM4_ESM.tif]

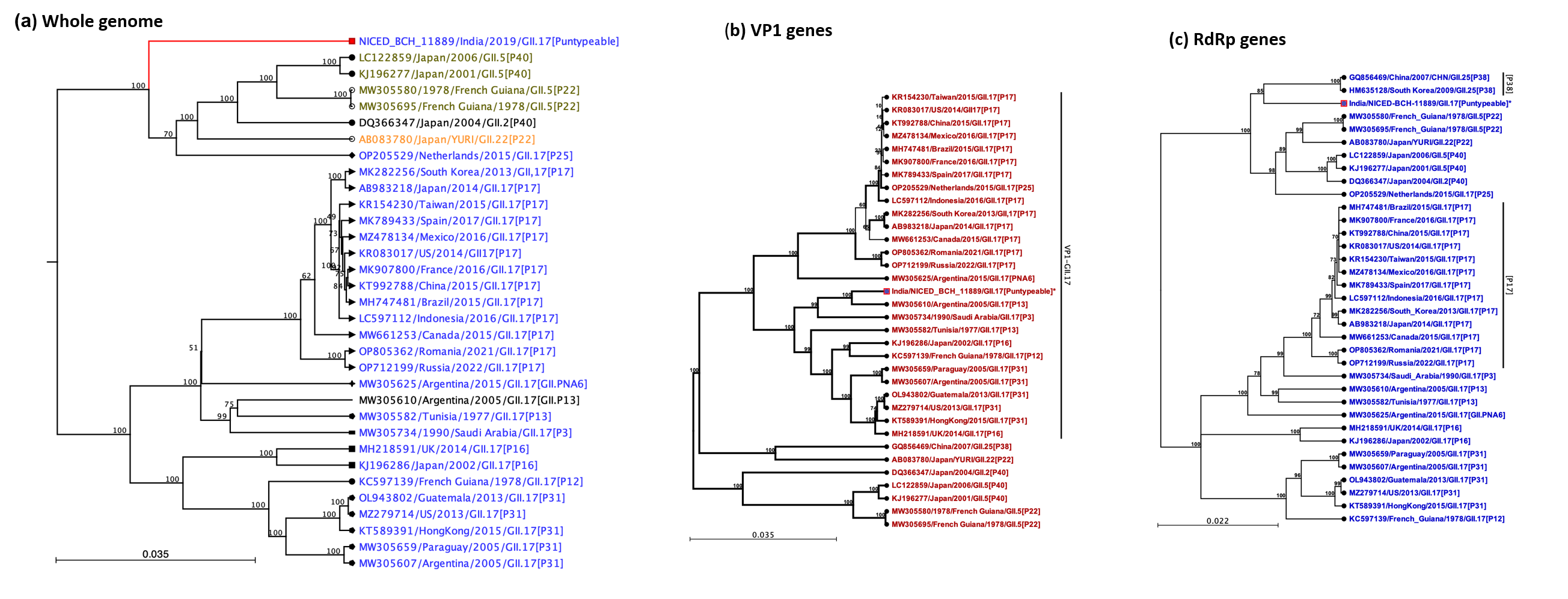

Supplement: Supplementary file 5 — Additional file 5: Figure S5. Phylogenetic tree of GII.17 and other strains. [file 13099_2023_594_MOESM5_ESM.tif]
