# Supplementary material for: Comprehensive full genome analysis of norovirus strains from eastern India, 2017–2021
Source: Gut Pathog. 2024 Jan 18;16:3. doi: 10.1186/s13099-023-00594-5 (PMC10797879; doi:10.1186/s13099-023-00594-5)
Supplement: Supplementary file 6 — Additional file 6: Table S1. Details of clinical samples enrolled in this study. [file 13099_2023_594_MOESM6_ESM.docx]

| **Sanmple ID** | **Age (months)** | **Sex** | **City/District** | **Hospitalisation status** | **Genotype** | **Vesikari score** | **Illness type** |
| --- | --- | --- | --- | --- | --- | --- | --- |
| **NICED-RV-100** | **18** | **M** | **Kolkata** | **Inpatients** | **GII.3[P16]** | **9** | **Moderate** |
| **NICED-RV-135** | **10** | **M** | **Kolkata** | **Inpatients** | **GII.4[P16]** | **10** | **Moderate** |
| **NICED-RV-281** | **10** | **F** | **Kolkata** | **Inpatients** | **GII.3[P16]** | **9** | **Moderate** |
| **NICED-RV-515** | **10** | **F** | **Kolkata** | **Inpatients** | **GII.4[P16]** | **9** | **Moderate** |
| **NICED-RV-567** | **9** | **M** | **Kolkata** | **Inpatients** | **GII.4[P16]** | **9** | **Moderate** |
| **NICED-RV-629** | **6** | **M** | **Kolkata** | **Inpatients** | **GII.3[P16]** | **8** | **Moderate** |
| **NICED-RV-659** | **9** | **M** | **Kolkata** | **Inpatients** | **GI.3[P13]** | **9** | **Moderate** |
| **NICED-RV-791** | **5** | **F** | **Kolkata** | **Inpatients** | **GI.3[P13]** | **8** | **Moderate** |
| **NICED-IDH-11808** | **24** | **M** | **Kolkata** | **Inpatients** | **GII.13[P16]** | **9** | **Moderate** |
| **NICED-IDH-11818** | **12** | **M** | **Kolkata** | **Inpatients** | **ND** | **10** | **Moderate** |
| **NICED-BCH-10206** | **11** | **M** | **Howrah** | **Outpatients** | **GII.4[P31]** | **10** | **Moderate** |
| **NICED-RV-987** | **9** | **F** | **Kolkata** | **Inpatients** | **GII.3[P16]** | **9** | **Moderate** |
| **NICED-RV-988** | **9** | **F** | **Kolkata** | **Inpatients** | **GII.3[P16]** | **9** | **Moderate** |
| **NICED-BCH-10486** | **4** | **M** | **Kolkata** | **Outpatients** | **GII.3[P16]** | **9** | **Moderate** |
| **NICED-RV-1218** | **18** | **F** | **Kolkata** | **Inpatients** | **GII.4[P16]** | **10** | **Moderate** |
| **NICED-BCH-10847** | **20** | **M** | **Kolkata** | **Outpatients** | **GII.4[P16]** | **9** | **Moderate** |
| **NICED-BCH-10861** | **11** | **M** | **Kolkata** | **Outpatients** | **GII.4[P16]** | **9** | **Moderate** |
| **NICED-BCH-10863** | **7** | **M** | **Kolkata** | **Outpatients** | **GII.4[P16]** | **9** | **Moderate** |
| **NICED-BCH-10876** | **12** | **M** | **South 24 Parganas** | **Outpatients** | **GI.3[P13]** | **8** | **Moderate** |
| **NICED-BCH-10892** | **11** | **F** | **North 24 Parganas** | **Outpatients** | **GI.3[P13]** | **9** | **Moderate** |
| **NICED-BCH-10957** | **18** | **M** | **South 24 Parganas** | **Outpatients** | **GII.4[P16]** | **10** | **Moderate** |
| **NICED-RV-1373** | **7** | **M** | **Kolkata** | **Inpatients** | **GII.3[P16]** | **10** | **Moderate** |
| **NICED-BCH-11123** | **10** | **M** | **South 24 Parganas** | **Outpatients** | **GII.3[P16]** | **9** | **Moderate** |
| **NICED-BCH-11157** | **11** | **M** | **Kolkata** | **Outpatients** | **GI.3[P13]** | **9** | **Moderate** |
| **NICED-BCH-11170** | **6** | **M** | **North 24 Parganas** | **Outpatients** | **GII.4[P16]** | **9** | **Moderate** |
| **NICED-BCH-11255** | **10** | **M** | **South 24 Parganas** | **Outpatients** | **GII.4[P16]** | **10** | **Moderate** |
| **NICED-BCH-11305** | **10** | **M** | **South 24 Parganas** | **Outpatients** | **GII.4[P16]** | **9** | **Moderate** |
| **NICED-BCH-11318** | **8** | **F** | **South 24 Parganas** | **Outpatients** | **ND** | **9** | **Moderate** |
| **NICED-BCH-11347** | **18** | **F** | **South 24 Parganas** | **Outpatients** | **ND** | **8** | **Moderate** |
| **NICED-BCH-11602** | **10** | **F** | **North 24 Parganas** | **Outpatients** | **GII.4[P16]** | **9** | **Moderate** |
| **NICED-BCH-11612** | **15** | **F** | **North 24 Parganas** | **Outpatients** | **GII.4[P16]** | **9** | **Moderate** |
| **NICED-BCH-11668** | **24** | **F** | **Kolkata** | **Outpatients** | **GII.4[P16]** | **10** | **Moderate** |
| **NICED-BCH-11710** | **11** | **M** | **North 24 Parganas** | **Outpatients** | **GII.4[P16]** | **10** | **Moderate** |
| **NICED-BCH-11725** | **12** | **M** | **North 24 Parganas** | **Outpatients** | **GII.4[P16]** | **9** | **Moderate** |
| **NICED-BCH-11726** | **9** | **F** | **Kolkata** | **Outpatients** | **GII.4[P16]** | **9** | **Moderate** |
| **NICED-BCH-11889** | **9** | **M** | **North 24 Parganas** | **Outpatients** | **GII.17[Put]** | **9** | **Moderate** |
| **NICED-BCH-12196** | **11** | **M** | **Kolkata** | **Outpatients** | **ND** | **9** | **Moderate** |
| **NICED-BCH-12197** | **9** | **F** | **North 24 Parganas** | **Outpatients** | **ND** | **10** | **Moderate** |
| **NICED-BCH-12243** | **10** | **M** | **South 24 Parganas** | **Outpatients** | **ND** | **9** | **Moderate** |
| **NICED-BCH-12332** | **30** | **F** | **Kolkata** | **Outpatients** | **ND** | **10** | **Moderate** |
| **NICED-BCH-12421** | **8** | **M** | **Kolkata** | **Outpatients** | **ND** | **9** | **Moderate** |
| **NICED-BCH-12621** | **8** | **M** | **Kolkata** | **Outpatients** | **GII.4[P31]** | **8** | **Moderate** |
| **NICED-BCH-12629** | **6** | **M** | **Kolkata** | **Outpatients** | **GII.16[P16]** | **8** | **Moderate** |
| **NICED-BCH-12650** | **5** | **F** | **Kolkata** | **Outpatients** | **ND** | **9** | **Moderate** |
| **NICED-BCH-12832** | **7** | **M** | **Kolkata** | **Outpatients** | **ND** | **9** | **Moderate** |

ND: Not detected by NGS

Additional file 6: Table S1. Details of clinical samples included in this study.
