# Supplementary material for: Comprehensive full genome analysis of norovirus strains from eastern India, 2017–2021
Source: Gut Pathog. 2024 Jan 18;16:3. doi: 10.1186/s13099-023-00594-5 (PMC10797879; doi:10.1186/s13099-023-00594-5)
Supplement: Supplementary file 7 — Additional file 7: Table S2. Alignment of unique informative amino acid sites in the of ORF-1 non-structural encoded proteins (NS1/2, NS4, NS6 and NS7) of GII[P16] genotype (after Barclay et al., 2019). Informative amino acid sites in the pre-2015 GII[P16] are labelled in light red, novel GII[P16] are labelled in green and Indian GII[P16] strains are labelled in blue. [file 13099_2023_594_MOESM7_ESM.docx]

|  | **Amino acid position**  **Strain names** | **NS 1/2** | | | | **NS4** | | | | | **NS6** | **NS7** | | | | |
| --- | --- | --- | --- | --- | --- | --- | --- | --- | --- | --- | --- | --- | --- | --- | --- | --- |
|  |  | **52** | **53** | **76** | **77** | **33** | **52** | **55** | **147** | **155** | **49** | **173** | **293** | **332** | **357** | **360** |
| **Pre-2015** | **MK752949.1/NoV/GII/Hu/US/2013/GII.2[P16]/Rock0558** | **N** | **P** | **-** | **-** | **R** | **K** | **S** | **P** | **V** | **V** | **D** | **S** | **V** | **K** | **T** |
|  | **MK754443.1/NoV/GII/Hu/US/2014/GII.3[P16]/Washington0967** | **N** | **S** | **-** | **-** | **R** | **K** | **T** | **P** | **A** | **V** | **D** | **S** | **V** | **K** | **T** |
|  | **MK753009.1/NoV/GII/Hu/US/2013/GII.3[P16]/Polk0892** | **N** | **S** | **-** | **-** | **R** | **K** | **T** | **P** | **A** | **V** | **D** | **S** | **V** | **K** | **T** |
|  | **MK764020.1/NoV/GII/Hu/US/2014/GII.3[P16]/Pittsylvania0388** | **N** | **P** | **-** | **-** | **R** | **K** | **I** | **P** | **A** | **V** | **D** | **S** | **I** | **K** | **A** |
|  | **MK753020.1/NoV/GII/Hu/US/2014/GII.13[P16]/HoodRiver0913** | **N** | **S** | **-** | **-** | **R** | **K** | **T** | **P** | **A** | **V** | **D** | **S** | **V** | **K** | **T** |
| **Post-2015** | **MK753034.1/NoV/GII/Hu/US/2017/GII.1[P16]/SaltLake0124** | **E** | **P** | **E** | **E** | **K** | **R** | **T** | **Q** | **T** | **I** | **E** | **T** | **I** | **Q** | **A** |
|  | **MK764022.1/NoV/GII/Hu/US/2018/GII.2[P16]/Hanover0423** | **K** | **P** | **E** | **E** | **K** | **R** | **T** | **Q** | **T** | **I** | **E** | **T** | **I** | **Q** | **A** |
|  | **MK773588.1/NoV/GII/Hu/US/2018/GII.3[P16]/Hennepin0275** | **E** | **P** | **E** | **E** | **K** | **R** | **T** | **Q** | **T** | **I** | **E** | **T** | **I** | **Q** | **A** |
|  | **MK764016.1/NoV/GII/Hu/US/2018/GII.4[P16]/Bay0244** | **K** | **S** | **E** | **E** | **K** | **R** | **T** | **Q** | **T** | **I** | **E** | **T** | **I** | **Q** | **A** |
|  | **MK754445.1/NoV/GII/Hu/US/2017/GII.12[P16]/Somerset0204** | **K** | **P** | **E** | **E** | **K** | **R** | **T** | **Q** | **T** | **I** | **E** | **T** | **I** | **Q** | **A** |
|  | **NC_039476.1/NoV/GIICHN/2016/GII.2[P16]/NCBI Ref seq** | **K** | **P** | **E** | **E** | **K** | **R** | **T** | **Q** | **T** | **I** | **E** | **T** | **I** | **Q** | **A** |
|  | **NC_039477.1/NoV/GII/GII.4[P16]/NCBI Ref seq** | **K** | **S** | **E** | **E** | **K** | **R** | **T** | **Q** | **T** | **I** | **E** | **T** | **I** | **Q** | **A** |
| **Indian GII[P16] strains** | **LC769707.1/NoV/GII/Hu/IND/2018/GII.4[P16]/NICED_RV_515** | **E** | **S** | **E** | **E** | **K** | **R** | **T** | **Q** | **T** | **I** | **E** | **T** | **I** | **Q** | **A** |
|  | **LC769708.1/NoV/GII/Hu/IND/2018/GII.4[P16]/NICED_RV_567(*)** | **E** | **P** | **E** | **E** | **K** | **R** | **T** | **Q** | **T** | **I** | **E** | **T** | **I** | **Q** | **A** |
|  | **LC769699.1/NoV/GII/Hu/IND/2019/GII.4[P16]/NICED_BCH_11726(*)** | **E** | **P** | **E** | **E** | **K** | **R** | **T** | **Q** | **T** | **I** | **E** | **T** | **I** | **Q** | **A** |
|  | **LC769698.1/NoV/GII/Hu/IND/2019/GII.4[P16]/NICED_BCH_11725(*)** | **E** | **P** | **E** | **E** | **K** | **R** | **T** | **Q** | **T** | **I** | **E** | **T** | **I** | **Q** | **A** |
|  | **LC769697.1/NoV/GII/Hu/IND/2019/GII.4[P16]/NICED_BCH_11710 (*)** | **E** | **L** | **E** | **E** | **K** | **R** | **T** | **Q** | **T** | **I** | **E** | **T** | **I** | **Q** | **A** |
|  | **LC769696.1/NoV/GII/Hu/IND/2019/GII.4[P16]/NICED_BCH_11668 (*)** | **E** | **P** | **E** | **E** | **K** | **R** | **T** | **Q** | **T** | **I** | **E** | **T** | **I** | **Q** | **A** |
|  | **LC769695.1/NoV/GII/Hu/IND/2019/GII.4[P16]/NICED_BCH_11612 (*)** | **E** | **P** | **E** | **E** | **K** | **R** | **T** | **Q** | **T** | **I** | **E** | **T** | **I** | **Q** | **A** |
|  | **LC769694.1/NoV/GII/Hu/IND/2019/GII.4[P16]/NICED_BCH_11602 (*)** | **E** | **P** | **E** | **E** | **K** | **R** | **T** | **Q** | **T** | **I** | **E** | **T** | **I** | **Q** | **A** |
|  | **LC769693.1/NoV/GII/Hu/IND/2019/GII.4[P16]/NICED_BCH_11305** | **E** | **P** | **E** | **E** | **K** | **R** | **T** | **Q** | **T** | **I** | **E** | **T** | **I** | **Q** | **A** |
|  | **LC769692.1/NoV/GII/Hu/IND/2019/GII.4[P16]/NICED_BCH_11255 (*)** | **E** | **P** | **E** | **E** | **K** | **R** | **T** | **Q** | **T** | **I** | **E** | **T** | **I** | **Q** | **A** |
|  | **LC769691.1/NoV/GII/Hu/IND/2019/GII.4[P16]/NICED_BCH_11170** | **E** | **P** | **E** | **E** | **K** | **R** | **T** | **Q** | **T** | **I** | **E** | **T** | **I** | **Q** | **A** |
|  | **LC769688.1/NoV/GII/Hu/IND/2019/GII.4[P16]/NICED_BCH_10957** | **E** | **P** | **E** | **E** | **K** | **R** | **T** | **Q** | **T** | **I** | **E** | **T** | **I** | **Q** | **A** |
|  | **LC769685.1/NoV/GII/Hu/IND/2019/GII.4[P16]/NICED_BCH_10863 (*)** | **E** | **P** | **E** | **E** | **K** | **R** | **T** | **Q** | **T** | **I** | **E** | **T** | **I** | **Q** | **A** |
|  | **LC769705.1/NoV/GII/Hu/IND/2017/GII.4[P16]/NICED_RV_135** | **E** | **P** | **E** | **E** | **K** | **R** | **T** | **Q** | **T** | **I** | **E** | **T** | **I** | **Q** | **A** |
|  | **LC769684.1/NoV/GII/Hu/IND/2019/GII.4[P16]/NICED_BCH_10861** | **E** | **P** | **E** | **E** | **K** | **R** | **T** | **Q** | **T** | **I** | **E** | **T** | **I** | **Q** | **A** |
|  | **LC769683.1/NoV/GII/Hu/IND/2019/GII.4[P16]/NICED_BCH_10847** | **E** | **P** | **E** | **E** | **K** | **R** | **T** | **Q** | **T** | **I** | **E** | **T** | **I** | **Q** | **A** |
|  | **LC769714.1/NoV/GII/Hu/IND/2019/GII.4[P16]/NICED_RV_1218** | **E** | **P** | **E** | **E** | **K** | **R** | **T** | **Q** | **T** | **I** | **E** | **T** | **I** | **Q** | **A** |
|  | **LC769715.1/NoV/GII/Hu/IND/2019/GII.3P16/NICED-RV-1373 (*)** | **E** | **P** | **E** | **E** | **K** | **R** | **T** | **Q** | **T** | **I** | **E** | **T** | **I** | **Q** | **A** |
|  | **LC769713.1/NoV/GII/Hu/IND/2018/GII.3P16/NICED-RV-988** | **E** | **P** | **E** | **E** | **K** | **R** | **T** | **Q** | **T** | **I** | **E** | **T** | **I** | **Q** | **A** |
|  | **LC769712.1/NoV/GII/Hu/IND/2018/GII.3P16/NICED-RV-987** | **E** | **P** | **E** | **E** | **K** | **R** | **T** | **Q** | **T** | **I** | **E** | **T** | **I** | **Q** | **A** |
|  | **LC769709.1/NoV/GII/Hu/IND/2018/GII.3P16/NICED-RV-629** | **E** | **P** | **E** | **E** | **K** | **R** | **T** | **Q** | **T** | **I** | **E** | **T** | **I** | **Q** | **A** |
|  | **LC769704.1/NoV/GII/Hu/IND/2017/GII.3P16/NICED-RV-100** | **E** | **P** | **E** | **E** | **K** | **R** | **T** | **Q** | **T** | **I** | **E** | **T** | **I** | **Q** | **A** |
|  | **LC769689.1/NoV/GII/Hu/IND/2019/GII.3P16/NICED-BCH-11123 (*)** | **E** | **P** | **E** | **E** | **K** | **R** | **T** | **Q** | **T** | **I** | **E** | **T** | **I** | **Q** | **A** |
|  | **LC769682.1/NoV/GII/Hu/IND/2019/GII.3P16/NICED-BCH-10486 (*)** | **E** | **P** | **E** | **E** | **K** | **R** | **T** | **Q** | **T** | **I** | **E** | **T** | **I** | **Q** | **A** |
|  | **LC769706.1/NoV/GII/Hu/IND/2017/GII.3P16/NICED-RV-281-ORF1** | **E** | **P** | **E** | **E** | **K** | **R** | **T** | **Q** | **T** | **I** | **E** | **T** | **I** | **Q** | **A** |
|  | **LC769703.1/NoV/GII/Hu/IND/2018/GII.13P16/NICED-IDH-11808** | **E** | **P** | **E** | **E** | **K** | **R** | **T** | **Q** | **T** | **V** | **E** | **T** | **I** | **Q** | **A** |
|  | **LC769702.1/NoV/GII/Hu/IND/2021/GII.16P16/NICED-BCH-12629** | **K** | **P** | **E** | **E** | **K** | **R** | **T** | **Q** | **T** | **I** | **E** | **T** | **I** | **Q** | **A** |

Additional file 7: Table S2: Alignment of unique informative amino acid sites in the of ORF-1 non-structural encoded proteins (NS1/2, NS4, NS6 and NS7) of GII[P16] genotype (after Barclay et al., 2019). Informative amino acid sites in the pre-2015 GII[P16] are labelled in light red, novel GII[P16] are labelled in green and Indian GII[P16] strains are labelled in blue.

* partial sequence is also available of these strains.
