# Supplementary material for: Comprehensive full genome analysis of norovirus strains from eastern India, 2017–2021
Source: Gut Pathog. 2024 Jan 18;16:3. doi: 10.1186/s13099-023-00594-5 (PMC10797879; doi:10.1186/s13099-023-00594-5)
Supplement: Supplementary file 8 — Additional file 8: Table S3. Alignment of antigenic epitope regions in the P2 subdomain of VP1 capsid protein of circulating GII.4 strains with vaccine strain GII.4c and different antigenic variants of GII.4 strains. [file 13099_2023_594_MOESM8_ESM.docx]

|  | **A** | | | | | | | | **C** | | | | | | | **D** | | | | | | | **E** | | | | | **F** | | **G** | | | | | | **I** | | | |
| --- | --- | --- | --- | --- | --- | --- | --- | --- | --- | --- | --- | --- | --- | --- | --- | --- | --- | --- | --- | --- | --- | --- | --- | --- | --- | --- | --- | --- | --- | --- | --- | --- | --- | --- | --- | --- | --- | --- | --- |
|  | **294** | **295** | **296** | **297** | **298** | **368** | **372** | **373** | **339** | **340** | **341** | **375** | **376** | **377** | **378** | **391** | **392** | **393** | **394** | **395** | **396** | **397** | **407** | **411** | **412** | **413** | **414** | **327** | **404** | **352** | **355** | **356** | **357** | **359** | **364** | **402** | **403** | **504** | **506** |
| **MK614455.1/Synthetic construct/ GII.4c/ TAK-214** | **A** | **G** | **T** | **Q** | **E** | **F** | **S** | **N** | **R** | **G** | **D** | **F** | **E** | **T** | **G** | **D** | **G** | **S** | **T** | **T** | **H** | **Q** | **N** | **R** | **D** | **S** | **H** | **V** | **V** | **S** | **S** | **V** | **H** | **T** | **S** | **Q** | **W** | **Q** | **D** |
| **AJ004864.1/Grimsby 1995** | **A** | **G** | **S** | **H** | **D** | **T** | **N** | **N** | **R** | **E** | **D** | **F** | **Q** | **T** | **G** | **D** | **G** | **N** | **N** | **H** | **Q** | **N** | **Y** | **T** | **G** | **H** | **N** | **V** | **V** | **S** | **S** | **V** | **H** | **I** | **S** | **Q** | **W** | **H** | **D** |
| **AY502023.1/Farmington Hills 2002** | **A** | **G** | **T** | **H** | **D** | **N** | **N** | **N** | **R** | **G** | **D** | **F** | **E** | **T** | **G** | **D** | **G** | **N** | **G** | **T** | **H** | **Q** | **S** | **R** | **T** | **G** | **H** | **V** | **V** | **S** | **D** | **V** | **H** | **T** | **S** | **Q** | **W** | **Q** | **D** |
| **EU078414.1/Hunter 2004** | **A** | **G** | **T** | **Q** | **N** | **S** | **S** | **N** | **R** | **R** | **D** | **F** | **E** | **T** | **G** | **D** | **G** | **S** | **T** | **T** | **H** | **Q** | **D** | **R** | **D** | **S** | **H** | **V** | **V** | **S** | **S** | **V** | **H** | **T** | **S** | **Q** | **W** | **Q** | **D** |
| **EF126963.1/Yerseke 2006** | **A** | **G** | **T** | **Q** | **E** | **S** | **S** | **N** | **R** | **R** | **D** | **F** | **E** | **T** | **G** | **D** | **G** | **S** | **T** | **T** | **H** | **Q** | **D** | **R** | **D** | **S** | **H** | **V** | **V** | **S** | **S** | **V** | **H** | **T** | **R** | **Q** | **W** | **Q** | **D** |
| **EF126965.1/Den Haag 2006** | **A** | **G** | **S** | **R** | **N** | **S** | **E** | **N** | **K** | **G** | **D** | **F** | **E** | **T** | **H** | **D** | **G** | **S** | **T** | **T** | **H** | **R** | **S** | **R** | **N** | **V** | **H** | **V** | **V** | **Y** | **S** | **A** | **P** | **T** | **S** | **Q** | **W** | **Q** | **D** |
| **AB434770.1/Osaka 2007** | **A** | **G** | **S** | **R** | **N** | **A** | **D** | **N** | **R** | **S** | **D** | **F** | **E** | **S** | **G** | **D** | **G** | **S** | **T** | **T** | **H** | **R** | **N** | **R** | **T** | **G** | **H** | **V** | **V** | **L** | **S** | **A** | **D** | **A** | **R** | **Q** | **W** | **Q** | **D** |
| **AB541274.1/Apeldoorn 2007** | **T** | **G** | **S** | **R** | **N** | **A** | **D** | **N** | **R** | **A** | **D** | **F** | **D** | **A** | **N** | **D** | **G** | **D** | **T** | **A** | **H** | **R** | **S** | **R** | **N** | **S** | **H** | **V** | **V** | **Y** | **S** | **A** | **D** | **A** | **R** | **Q** | **W** | **Q** | **D** |
| **GU445325.2/New Orleans 2009** | **P** | **G** | **S** | **R** | **N** | **A** | **D** | **N** | **R** | **T** | **N** | **F** | **E** | **T** | **N** | **D** | **G** | **S** | **T** | **T** | **P** | **R** | **S** | **R** | **N** | **I** | **H** | **V** | **V** | **Y** | **S** | **A** | **D** | **S** | **R** | **Q** | **W** | **Q** | **D** |
| **MN400355.2/Hong Kong 2019** | **A** | **G** | **T** | **R** | **Q** | **G** | **E** | **D** | **K** | **G** | **D** | **L** | **Q** | **S** | **G** | **D** | **G** | **E** | **N** | **P** | **H** | **F** | **S** | **R** | **T** | **G** | **H** | **V** | **V** | **S** | **A** | **A** | **D** | **A** | **N** | **Q** | **W** | **P** | **D** |
| **JX459908.1/Sydney 2012** | **T** | **G** | **S** | **R** | **N** | **E** | **D** | **R** | **R** | **T** | **D** | **F** | **E** | **A** | **N** | **D** | **G** | **G** | **T** | **T** | **H** | **R** | **S** | **R** | **N** | **T** | **H** | **V** | **V** | **Y** | **S** | **A** | **D** | **A** | **R** | **Q** | **W** | **Q** | **D** |
| **LC175468.1/Sydney 2012** | **T** | **G** | **S** | **R** | **N** | **E** | **D** | **H** | **R** | **T** | **D** | **F** | **E** | **A** | **N** | **D** | **G** | **S** | **T** | **T** | **H** | **R** | **S** | **R** | **N** | **T** | **H** | **V** | **V** | **Y** | **S** | **A** | **D** | **A** | **R** | **Q** | **W** | **Q** | **D** |
| **NC_039477.1/Sydney 2012** | **T** | **G** | **S** | **R** | **N** | **E** | **D** | **H** | **R** | **T** | **D** | **F** | **E** | **A** | **N** | **D** | **G** | **S** | **T** | **T** | **H** | **R** | **S** | **R** | **N** | **T** | **H** | **V** | **V** | **Y** | **S** | **A** | **D** | **A** | **R** | **Q** | **W** | **Q** | **D** |
| **Cluster A (n=6)** | **T** | **G** | **S** | **R** | **N** | **E** | **D** | **H** | **R** | **T** | **D** | **F** | **E** | **A** | **N** | **D** | **G** | **S** | **T** | **T** | **H** | **R** | **S** | **R** | **N** | **T** | **H** | **V** | **V** | **Y** | **S** | **A** | **D** | **A** | **R** | **Q** | **W** | **Q** | **D** |
| **Cluster B (n=5)** | **T** | **G** | **S** | **H** | **N** | **E** | **N** | **H** | **R** | **T** | **D** | **F** | **E** | **V** | **N** | **D** | **G** | **S** | **T** | **T** | **H** | **R** | **S** | **R** | **N** | **T** | **H** | **V** | **V** | **Y** | **S** | **A** | **D** | **A** | **R** | **Q** | **W** | **Q** | **D** |
| **Cluster C (n=2)** | **T** | **G** | **S** | **H** | **N** | **E** | **N** | **H** | **R** | **T** | **D** | **F** | **E** | **A** | **N** | **D** | **G** | **S** | **T** | **T** | **H** | **R** | **S** | **R** | **N** | **T** | **H** | **V** | **V** | **Y** | **S** | **A** | **D** | **A** | **R** | **Q** | **W** | **Q** | **D** |
| **LC769697/NICED_BCH_11710** | **A** | **G** | **S** | **R** | **N** | **E** | **D** | **H** | **R** | **T** | **D** | **F** | **E** | **V** | **N** | **D** | **G** | **G** | **T** | **T** | **H** | **R** | **S** | **R** | **N** | **T** | **H** | **V** | **V** | **Y** | **S** | **A** | **D** | **A** | **R** | **Q** | **W** | **Q** | **D** |
| **LC769695/NICED_BCH_11612** | **T** | **G** | **S** | **H** | **N** | **E** | **N** | **N** | **R** | **T** | **D** | **F** | **E** | **A** | **N** | **D** | **G** | **G** | **T** | **T** | **H** | **R** | **S** | **R** | **N** | **T** | **H** | **V** | **V** | **Y** | **S** | **A** | **D** | **A** | **R** | **Q** | **W** | **Q** | **D** |
| **LC769693/NICED_BCH_11305** | **T** | **G** | **S** | **H** | **N** | **E** | **N** | **H** | **R** | **T** | **D** | **F** | **E** | **A** | **N** | **D** | **G** | **G** | **T** | **T** | **H** | **R** | **S** | **R** | **N** | **T** | **H** | **V** | **V** | **Y** | **S** | **A** | **D** | **A** | **R** | **Q** | **W** | **Q** | **D** |
| **LC769707/NICED_RV_515** | **T** | **G** | **S** | **H** | **N** | **E** | **N** | **H** | **R** | **T** | **D** | **F** | **E** | **V** | **N** | **D** | **G** | **G** | **T** | **T** | **H** | **R** | **S** | **R** | **N** | **T** | **H** | **V** | **V** | **Y** | **S** | **A** | **D** | **A** | **R** | **Q** | **W** | **Q** | **D** |
| **LC769701/NICED_BCH_12621** | **T** | **G** | **T** | **H** | **N** | **E** | **N** | **H** | **R** | **G** | **D** | **F** | **E** | **T** | **N** | **N** | **G** | **D** | **T** | **A** | **H** | **R** | **S** | **R** | **N** | **T** | **H** | **V** | **V** | **Y** | **S** | **N** | **D** | **T** | **R** | **Q** | **W** | **Q** | **D** |
| **LC769681/NICED_BCH_10206** | **T** | **G** | **S** | **H** | **N** | **E** | **N** | **H** | **R** | **G** | **D** | **F** | **E** | **T** | **N** | **N** | **G** | **D** | **T** | **A** | **H** | **R** | **S** | **R** | **N** | **T** | **H** | **V** | **V** | **Y** | **S** | **N** | **D** | **T** | **R** | **Q** | **W** | **Q** | **D** |

**Additional file 8: Table S3.** Alignment of antigenic epitope regions in the P2 subdomain of VP1 capsid protein of circulating GII.4 Sydney strains with vaccine strain GII.4c

and different antigenic variants of GII.4 strains.

**Cluster A**= LC769705/NICED_RV_135, LC769708/NICED_RV_567, LC769696/NICED_BCH_11668, LC769694/NICED_BCH_11602, LC769691/NICED_BCH_11170, LC769688/NICED_BCH_10957

**Cluster B**= LC769714/NICED_RV_1218, LC769692/ NICED_BCH_11255, LC769685/NICED_BCH_10863, LC769684/NICED_BCH_10861, LC769683/NICED_BCH_10847

**Cluster C** = LC769698/NICED_BCH_11725, LC769699/NICED_BCH_11726.

**Keys: Orange** Small nonpolar (G, A, S, T); **Green** Hydrophobic (C, V, I, L, P, F, Y, M, W); **Magenta** Polar (N, Q, H); **Red** Negatively charged (D, E); **Blue** Positively charged (K, R)
