# Supplementary material for: Comprehensive full genome analysis of norovirus strains from eastern India, 2017–2021
Source: Gut Pathog. 2024 Jan 18;16:3. doi: 10.1186/s13099-023-00594-5 (PMC10797879; doi:10.1186/s13099-023-00594-5)
Supplement: Supplementary file 9 — Additional file 9: Table S4. Analysis of amino acid sequences in the HBGA binding pockets of P domain in GII.4 capsid proteins. (Completely conserved or mostly conserved regions are highlighted in dark red and variable amino acid residues are highlighted in yellow). [file 13099_2023_594_MOESM9_ESM.docx]

**Additional file 9: Table S4:** Analysis of amino acid sequences in the HBGA binding pockets of P domain in GII.4 capsid proteins. (Completely conserved or mostly conserved regions are highlighted in dark red and variable amino acid residues are highlighted in yellow).

| **Strain names** | **β-sheet**  **(residues**  **341–346)** | **Loop 1**  **(residues**  **372-378)** | **Loop 2**  **(residues**  **390–396)** | **Loop 3**  **(residues 441–445)** |
| --- | --- | --- | --- | --- |
| **AJ004864.1\|GII.4_Grimsby_1995** | **DGSTRA** | **NNDFQTG** | **QDGN­­­-NH** | **CSGYP** |
| **AY502023.1\|GII.4_Farmington_2002** | **DGSTRG** | **NNDFETG** | **QDGNGTH** | **CSGYP** |
| **EU078414.1\|GII.4_Hunter_2004** | **DGSTRG** | **SNDFETG** | **QDGSTTH** | **CSGYP** |
| **EF126963.1\|GII.4_Yerseke_2006** | **DGSTRG** | **SNDFETG** | **QDGSTTH** | **CSGYP** |
| **EF126965.1\|GII.4_Den Haag_2006** | **DGSTRG** | **ENDFETH** | **QDGSTTH** | **CSGYP** |
| **AB434770.1\|GII.4_Osaka_2007** | **DGSTRG** | **DNDFESG** | **QDGSTTH** | **CSGYP** |
| **AB541274.1\|GII.4_Apeldoorn_2007** | **DGSTRG** | **DNDFDAN** | **QDGDTAH** | **CSGYP** |
| **GU445325.2\|GII.4_New Orleans_2009** | **NGSTRG** | **DNDFETN** | **QDGSTTP** | **CSGYP** |
| **JX459908.1\|GII.4[P31]_Sydney_2012** | **DGSTRG** | **DRDFEAN** | **QDGGTTH** | **CSGYP** |
| **NC_039477.1\|GII.4[P16]_Sydney_2012** | **DGSTRG** | **DHDFEAN** | **QDGSTTH** | **CSGYP** |
| **LC175468.1\|GII.4[P16]_Sydney_2012** | **DGSTRG** | **DHDFEAN** | **QDGSTTH** | **CSGYP** |
| **MN400355.2\|GII.4_Hong Kong_2019** | **DGSTRG** | **EDDLQSG** | **QDGENPH** | **CSGYP** |
| **Group A (n=6)** | **DGSTRG** | **DHDFEAN** | **QDGSTTH** | **CSGYP** |
| **Group B (n=5)** | **DGSTRG** | **NHDFEVN** | **QDGSTTH** | **CSGYP** |
| **Group C (n=4)** | **DGSTRG** | **NNDFEAN** | **QDGGTTH** | **CSGYP** |
| **Group D (n=2)** | **DGSTRG** | **NHDFETN** | **QNGDTAH** | **CSGYP** |
| **NICED_RV_515** | **DGSTRG** | **NHDFEVN** | **QDGGTTH** | **CSGYP** |
| **NICED_BCH_11710** | **DGSTRG** | **DHDFEVN** | **QDGGTTH** | **CSGYP** |

**Group A:** NICED_RV_135; NICED_RV_567; NICED_BCH_10957; NICED_BCH_11170; NICED_BCH_11602; NICED_BCH_11668

**Group B:** NICED_RV_1218; NICED_BCH_10847; NICED_BCH_10861; NICED_BCH_10863; NICED_BCH_11255

**Group C:** NICED_BCH_11612; NICED_BCH_11726; NICED_BCH_11725; NICED_BCH_11305

**Group D:** NICED_BCH_12621; NICED_BCH_10206
